# The genetic basis of natural antibody titers of young healthy pigs and relationships with disease resilience

**DOI:** 10.1186/s12864-020-06994-0

**Published:** 2020-09-22

**Authors:** Yulu Chen, Laura E. Tibbs-Cortes, Carolyn Ashley, Austin M. Putz, Kyu-Sang Lim, Michael K. Dyck, Frederic Fortin, Graham S. Plastow, Jack C. M. Dekkers, John C. S. Harding, PigGen Canada, Pig Gen Canada

**Affiliations:** 1 Department of Animal Science, Iowa State University, Ames, IA USA; 2 Department of Agronomy, Iowa State University, Ames, IA USA; 3 Department of Large Animal Clinical Sciences, University of Saskatchewan, Saskatoon, SK Canada; 4 Department of Agriculture, Food and Nutritional Science, University of Alberta, Edmonton, AB Canada; 5 Centre de développement du porc du Québec inc. (CDPQ), Québec City, QC Canada

**Keywords:** Disease resilience, Natural antibody, Genetic parameter, Polymicrobial disease challenge, GWAS

## Abstract

**Background:**

Disease resilience is the ability to maintain performance under pathogen exposure but is difficult to select for because breeding populations are raised under high health. Selection for resilience requires a trait that is heritable, easy to measure on healthy animals, and genetically correlated with resilience. Natural antibodies (NAb) are important parts of the innate immune system and are found to be heritable and associated with disease susceptibility in dairy cattle and poultry. Our objective was to investigate NAb and total IgG in blood of healthy, young pigs as potential indicator traits for disease resilience.

**Results:**

Data were from Yorkshire x Landrace pigs, with IgG and IgM NAb (four antigens) and total IgG measured by ELISA in blood plasma collected ~ 1 week after weaning, prior to their exposure to a natural polymicrobial challenge. Heritability estimates were lower for IgG NAb (0.12 to 0.24, + 0.05) and for total IgG (0.19 + 0.05) than for IgM NAb (0.33 to 0.53, + 0.07) but maternal effects were larger for IgG NAb (0.41 to 0.52, + 0.03) and for total IgG (0.19 + 0.05) than for IgM NAb (0.00 to 0.10, + 0.04). Phenotypically, IgM NAb titers were moderately correlated with each other (average 0.60), as were IgG NAb titers (average 0.42), but correlations between IgM and IgG NAb titers were weak (average 0.09). Phenotypic correlations of total IgG were moderate with NAb IgG (average 0.46) but weak with NAb IgM (average 0.01). Estimates of genetic correlations among NAb showed similar patterns but with small SE, with estimates averaging 0.76 among IgG NAb, 0.63 among IgM NAb, 0.17 between IgG and IgM NAb, 0.64 between total IgG and IgG NAb, and 0.13 between total IgG and IgM NAb. Phenotypically, pigs that survived had slightly higher levels of NAb and total IgG than pigs that died. Genetically, higher levels of NAb tended to be associated with greater disease resilience based on lower mortality and fewer parenteral antibiotic treatments. Genome-wide association analyses for NAb titers identified several genomic regions, with several candidate genes for immune response.

**Conclusions:**

Levels of NAb in blood of healthy young piglets are heritable and potential genetic indicators of resilience to polymicrobial disease.

## Background

The swine industry suffers enormous economic losses as a result of infectious diseases. The porcine reproductive and respiratory syndrome virus (PRRSV), for example, costs the U.S. swine industry about $664 million a year [1]. Over the past decade, attention to the genetics of health traits in pigs is increasing because reducing the economic losses brought on by diseases can greatly improve the production efficiency of pork, along with improving animal welfare and reducing use of antibiotics. Selection for disease resilience, which refers to the ability of an animal to maintain performance when exposed to disease [2–5], can be an effective strategy to reduce the impact of disease [6]. However, selecting for disease resilience is difficult because nucleus breeding stock are typically kept in high-health environments. Therefore, the development of indicators of disease resilience that can be measured on young, healthy pigs may lead to practical methods to select for disease resilience. For an indicator trait to be effective for genetic improvement of disease resilience, it must be heritable, easy to measure at a young age in a high-health environment, and genetically correlated with disease resilience.

Natural antibodies (NAb) are produced without prior infection, foreign antigen exposure, or passive immunization [7–9], and have been shown to be present in blood and milk without prior exposure to pathogens [10, 11]. NAb are poly-reactive antibodies that bind different pathogen-associated molecular patterns (PAMPs), are part of the innate immune system, and play important roles in the first line of defense against pathogens [12]. PAMPs are a set of microbial molecules that are shared among related pathogens but vary between groups of pathogens, including glycans and glycoconjugates [13]. The innate immune system recognizes PAMPs to activate immune responses and protect the host. NAb levels have been measured in vitro using important PAMPs [10, 14–17], including: lipopolysaccharides (LPS), which are endotoxins found on the cell membranes of gram-negative bacteria, such as *Escherichia coli* and *Salmonella* spp. [18]; lipoteichoic acid (LTA), which is present in Gram-positive bacteria, such as *Staphylococcus aureus* [19]; peptidoglycan (PDG), which is a polymer that consists of sugars and amino acids that form a mesh-like layer outside the plasma membrane of most bacteria, but with the proportion of PDG being high (90%) in Gram-positive bacteria and low (10%) in Gram-negative bacteria [20]; and keyhole limpet hemocyanin (KLH), which is a large, copper-containing and oxygen-carry protein molecule derived from the hemolymph of the inedible mollusc, *Megathura crenulate* [21]. NAb have different isotypes, of which IgM is most common, with IgG and IgA being less abundant [7].

Several studies have shown the level of NAb in blood or milk to be repeatable and heritable. Ploegaert et al. [11, 14] showed that the level of NAb was repeatable within the same cow but differed between cows. NAb levels in blood and milk were found to be heritable in dairy cattle, with estimates ranging from 0.18 to 0.45 for IgM isotypes, and from 0.08 to 0.31 for IgG isotypes [14, 22–24]. Estimates of the heritability of NAb titers were higher in blood (0.15 to 0.25) than in milk (0.08 to 0.23) [14, 22–24]. Wijga et al. [25] and Berghof et al. [15] estimated the heritability of total NAb to range from 0.12 to 0.23 in poultry.

Several studies in dairy cattle and laying hens have investigated the potential of NAb as indicators of disease resistance or resilience. In dairy cattle, low levels of NAb in plasma against KLH and LPS tended to be phenotypically associated with better metabolic health, but these associations were not significant [10]. Knegsel et al. [26] showed that clinical mastitis incidence tended to be phenotypically positively correlated with the level of NAb binding myosin (*P* = 0.06) and negatively with NAb binding transferrin (*P* = 0.08). Thompson-Crispi et al. [24] showed that dairy cows that have a high level of IgM NAb had a tendency to have the lowest incidence rate of clinical mastitis. In poultry, Star et al. [27] found that the levels of NAb binding KLH and LPS were associated with survival of chickens. Berghof et al. [28] found that selective breeding for high KLH NAb levels at 16 weeks of age increased resistance to avian pathogenic *Escherichia coli* (APEC) in early life and reported that there was a potential for KLH-binding NAb to be used as an indicator trait to select for general disease resistance in poultry.

In a genome-wide association study (GWAS) in Canadian Holstein cows, Klerk et al. [16] identified several Single Nucleotide Polymorphisms (SNPs) that were associated with IgG NAb titers in blood, with nearby candidate genes that were related to the biological function of NAb. Berghof et al. [17] identified one genomic region on chromosome 4 that was associated with KLH-binding IgM NAb titers in blood of chickens, which included the toll-like receptor 1 family member B gene (*TLR1B*).

In 2015, a natural disease challenge model (NDCM) was established in Deschambault, Québec, Canada, to investigate potential indicators of disease resilience in pigs. One objective of this project was to investigate a number of immune response measures, including NAb and total IgG, as potential biomarkers for disease resilience that could be measured in healthy animals pre-challenge, as would occur if included in genetic selection programs in high-health nucleus farms. The specific objective of the present study was to use data from this project to investigate the genetic basis of the levels of NAb in blood from young healthy pigs and their use as potential indicator traits for disease resilience.

## Results

### Antibody levels

Nine antibody assays were conducted on blood samples collected on 1704 pigs, 5 days after arrival in the quarantine nursery, at approximately 27 days of age. NAb levels for isotypes IgG and IgM binding KLH (KLH-G, KLH-M), LPS (LPS-G, LPS-M), LTA (LTA-G, LTA-M), and PGN (PDG-G, PDG-M) were measured, as well as total IgG (IgG-T). NAb levels were reported as sample positive ratios (S/P), based on a positive control sample that was specific to each assay. Table 1 shows descriptive statistics and Supplementary Figure 1 shows distributions of antibody levels and of the natural logarithm of antibody levels. Log transformation resulted in close to normal distributions and was used for data analysis.

**Table 1:** Descriptive statistics of log_e_-transformed sample/positive ratio (S/P) for IgG and IgM natural antibodies against four antigens and for total IgG in blood of young healthy pigs, and of their subsequent performance and resilience phenotypes during a natural polymicrobial disease challenge

Trait	N^p^	Mean^q^	SD^r^	Min^s^	Max^t^
KLH^a^-G^h^	1704	− 1.13	0.61	−3.56	0.79
LPS^b^-G	1702	−1.01	0.62	−3.58	0.85
LTA^c^-G	1704	−0.50	0.77	−3.40	1.10
PDG^d^-G	1704	−0.37	0.37	−2.30	0.55
Ave-G^e^	1702	−0.75	0.43	−2.99	1.15
KLH-M^i^	1702	−0.26	0.62	−2.62	1.25
LPS-M	1703	−0.27	0.62	−2.66	1.67
LTA-M	1703	−1.77	0.88	−4.39	0.79
PDG-M	1702	−0.46	0.56	−2.62	0.94
Ave-M^f^	1699	−0.69	0.57	−2.99	0.91
IgG-T^g^	1640	−0.33	0.47	−3.03	1.15
ALLMOR^j^	3286	0.26	0.44	0.00	1.00
cNurMOR^k^	3256	0.11	0.32	0.00	1.00
FinMOR^l^	2886	0.16	0.37	0.00	1.00
cNurTRT^m^	3231	1.17	1.23	0.00	10.50
FinTRT^n^	2375	0.34	0.62	0.00	4.79
ALLTRT^o^	2941	2.02	2.13	0.00	15.32
qNurADG^p^	3277	0.28	0.10	−0.21	0.58
cNurADG^q^	3246	0.29	0.18	−0.31	1.00
cFinADG^r^	2376	0.90	0.13	0.27	1.25
ADFI^s^	2376	2.20	0.32	0.92	3.19
VAR_FI_^t^	2464	0.50	0.10	0.19	0.95
VAR_DUR_^u^	2464	13.48	3.91	6.22	34.55
OFF_FI_^v^	2463	0.04	0.07	0.00	0.87
OFF_DUR_^w^	2463	0.04	0.05	0.00	0.49

^a^KLH = keyhole limpet hemocyanin

^b^LPS = lipopolysaccharide

^c^LTA = lipoteichoic acid

^d^PDG = peptidoglycan

^e^Ave-G = the average NAb IgG isotype binding to the four antigens

^f^Ave-M = the average NAb IgM isotype binding to the four antigens

^g^IgG-T = total IgG level in plasma (unit is mg/ml)

^h^G = isotype IgG binding to the four antigens

^i^M = isotype IgM binding to the four antigens

^j^ALLMOR = 1 for pigs that that died prior to slaughter; = 0 for pigs that survived to slaughter

^k^cNurMOR = mortality for challenge nursery period

^l^FinMOR = mortality for challenge finishing period

^m^cNurTRT = the number of treatment rate adjusted to 27 days during the challenge nursery in the NCDM

^n^FinTRT = the number of treatment rate adjusted to 100 days during the finishing period in the NCDM

^o^ALLTRT = the number of treatment rate adjusted to 180 days in the NCDM

^p^qNurADG = average daily gain for quarantine nursery period

^q^cNurADG = average daily gain for challenge nursery period

^r^cFinADG = average daily gain for challenge finishing period

^s^ADFI = average daily feed intake

^t^VAR_FI_ = the root mean square error from the within individual regression of feed intake on age

^u^VAR_DUR_ = the root mean square error from the within individual regression of feed intake duration on age

^v^OFF_FI_ = the proportion of negative residuals for 5% quantile regression of feed intake on age across all pigs

^w^OFF_DUR_ = the proportion of negative residuals for 5% quantile regression of feed intake duration on age across all pigs

Table 2 shows heritability estimates and proportions of variance due to litter (maternal) effects for individuals NAb levels and of the average of the natural logarithm of IgG and IgM NAb levels across antigens. Estimates of heritability were lower for IgG NAb than for IgM NAb but the opposite was observed for the proportion of variance explained by litter. Estimates of heritability and litter effects for IgG-T were both 0.19.

**Table 2:** Estimates of heritability and of the proportion of phenotypic variance explained by litter effects for log_e_-transformed sample/positive ratios for IgG and IgM natural antibody against four antigens and for total IgG in blood of young healthy piglets

Antibody	Heritability (*SE*)	Litter effects (*SE*)
KLH^a^-G^e^	0.13 (0.05)	0.46 (0.03)
LPS^b^-G	0.14 (0.05)	0.53 (0.03)
LTA^c^-G	0.18 (0.05)	0.56 (0.03)
PDG^d^-G	0.15 (0.05)	0.51 (0.03)
Ave-G^g^	0.23 (0.05)	0.43 (0.03)
KLH-M^f^	0.36 (0.07)	0.10 (0.04)
LPS-M	0.45 (0.07)	0.06 (0.03)
LTA-M	0.54 (0.06)	0.00 (0.00)
PDG-M	0.36 (0.06)	0.08 (0.03)
Ave-M^h^	0.51 (0.07)	0.04 (0.03)
IgG-T^i^	0.19 (0.06)	0.19 (0.04)

^a^KLH = keyhole limpet hemocyanin

^b^LPS = lipopolysaccharide

^c^LTA = lipoteichoic acid

^d^PDG = peptidoglycan

^e^G = isotype IgG

^f^M = isotype IgM

^g^Ave-G = the average of log_e_NAb IgG across the four antigens

^h^Ave-M = the average of log_e_NAb IgM across the four antigens

^i^IgGT = the S/P ratio for total IgG level in plasma

**Fig. 1:**
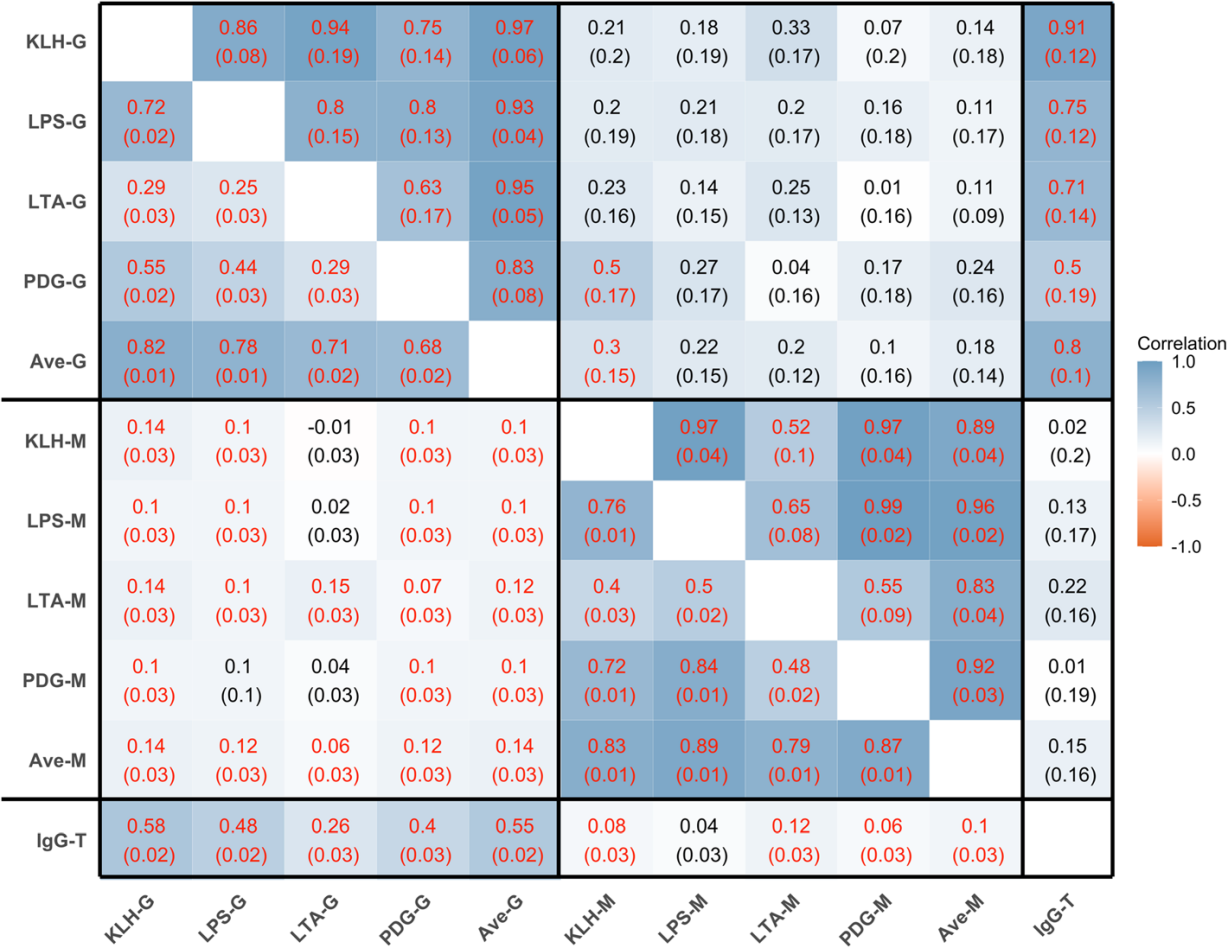
Estimates of genetic (above diagonal) and phenotypic (below diagonal) correlations (SE) for log_e_-transformed sample/positive ratios for IgG and IgM natural antibodies against four antigens and for total IgG in blood of young healthy piglets. Red font means the correlation is significant (*p*-value is less than 0.05 based on z test). KLH = keyhole limpet hemocyanin. LPS = lipopolysaccharide. LTA = lipoteichoic acid. PDG = peptidoglycan. Ave-G = the average NAb IgG isotype binding to the four antigens. Ave-M_ave = the average NAb IgM isotype binding to the four antigens. IgG-T = total IgG level in plasma (unit is mg/ml). G = isotype IgG binding to the four antigens. M = isotype IgM binding to the four antigens

Estimates of genetic and phenotypic correlations among NAb and with IgG-T are in Fig. 1. Phenotypically, IgG NAb titers were correlated with each other (average correlation of 0.55, ranging from 0.25 to 0.82), as were IgM NAb levels (average correlation of 0.71, ranging from 0.40 to 0.89), but correlations between the IgM and IgG NAb levels were close to zero (average correlation of 0.10, ranging from − 0.01 to 0.14). Estimates of genetic correlations among antibody titers showed similar patterns as the phenotypic correlations but tended to be stronger, averaging 0.85 (0.75 to 0.97) among IgG NAb, 0.83 (0.52 to 0.99) among IgM NAb, and 0.19 (0.01 to 0.50) between IgG and IgM NAb. Figure 1 also shows estimates of the genetic and phenotypic correlations of the level of IgG-T with NAb levels, which were stronger for NAb IgG than for NAb IgM, both phenotypically (averaging 0.45 for IgG NAb, ranging from 0.26 to 0.58, and averaging 0.08 for IgM NAb, ranging from 0.04 to 0.12) and genetically (averaging 0.73 for IgG NAb, ranging from 0.50 to 0.91, and averaging 0.11 for IgM NAb, ranging from 0.01 to 0.22).

### Relationships with performance and resilience under disease

Performance and resilience phenotypes were collected in the challenge nursery and finisher, as described by Putz et al. [6]: mortality (1 for pigs that that died prior to slaughter; 0 for pigs that survived to slaughter) for the whole challenge period (ALLMOR), and while in the challenge nursery (cNurMOR) or finisher (FinMOR); number of individual parenteral antibiotic treatments adjusted to 180 days (average age at slaughter) during the entire challenge period (ALLTRT), to 27 days during the challenge nursery period (cNurTRT), and to 100 days during the finishing period (FinTRT); average daily gain (ADG) in the quarantine nursery (qNurADG), in the challenge nursery (cNurADG), and in the finish (cFinADG); average daily feed intake in the finisher (ADFI); day-to-day variation in feed intake or duration in the finisher based on the root mean square error of the regression of daily feed intake (VAR_FI_) or daily feed intake duration (VAR_DUR_) on age; and the proportion of off-feed days in the finisher based on the proportion on negative residuals for 5% quantile regression of daily feed intake (OFF_FI_) or daily feed intake duration (OFF_DUR_) on age across all animals. The RMSE and QRP traits were based on Putz et al. [6], who found that susceptible animals tended to have higher RMSE and QRP than resilient animals.

**Fig. 2:**
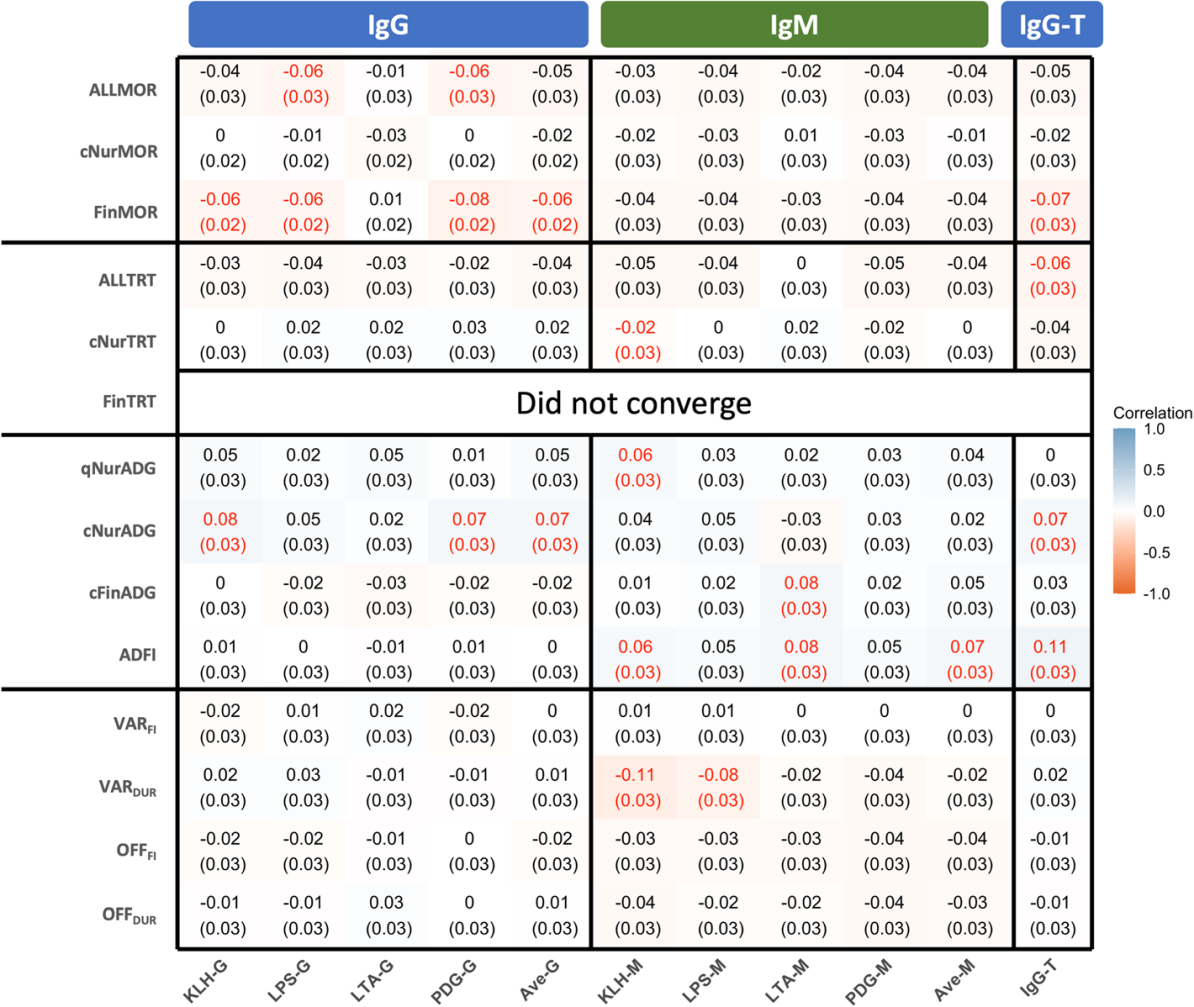
Estimates of phenotypic correlations of log_e_-transformed sample/positive ratios for IgG and IgM natural antibodies against four antigens and for total IgG in blood of young healthy piglets with their subsequent performance and resilience phenotypes during a natural polymicrobial disease challenge. Red font means the correlations is significant (*p*-value is less than 0.05 based on z test). KLH = keyhole limpet hemocyanin. LPS = lipopolysaccharide. LTA = lipoteichoic acid. PDG = peptidoglycan. Ave-G = the average NAb IgG isotype binding to the four antigens. Ave-M = the average NAb IgM isotype binding to the four antigens. IgG-T = total IgG level in plasma (unit is mg/ml). G = isotype IgG binding to the four antigens. M = isotype IgM binding to the four antigens. ALLMOR = 1 for pigs that that died prior to slaughter; = 0 for pigs that survived to slaughter. cNurMOR = mortality for challenge nursery period. FinMOR = mortality for challenge finishing period. ALLTRT = treat rate adjusted to 180 days. qNurADG = average daily gain for quarantine nursery period. cNurADG = average daily gain for challenge nursery period. cFinADG = average daily gain for challenge finishing period. ADFI = average daily feed intake. VAR_FI_ = the root mean square error from the within individual regression of feed intake on age. VAR_DUR_ = the root mean square error from the within individual regression of feed intake duration on age. OFF_FI_ = the proportion of negative residuals for 5% quantile regression of feed intake on age across all pigs. OFF_DUR_ = the proportion of negative residuals for 5% quantile regression of feed intake duration on age across all pigs

Descriptive statistics for the analyzed resilience traits are shown in Table 1. Figure 2 shows estimates of phenotypic correlations of antibody levels with performance and resilience traits. Overall, phenotypic correlations of antibody levels with performance and resilience traits were weak, but some correlations were significant, including a negative association of NAb and IgG-T with ALLMOR and nTrtPer180.

Table 3 shows estimates of phenotypic associations of NAb and IgG-T with subsequent mortality based on the difference in NAb or IgG-T for pigs that died versus those that survived. Pigs that died in the challenge nursery and/or the finisher had lower NAb and IgG-T levels than pigs that survived. However, these differences were only significant for the IgG NAb isotype and IgG-T during the finisher period and during the entire challenge period.

**Table 3:** Estimates of the phenotypic difference of log_e_-transformed sample/positive ratios for IgG and IgM natural antibodies against four antigens and for total IgG between pigs that subsequently died versus survived in the nursery and/or finisher following a natural polymicrobial disease challenge

	Nursery		Finisher		Nursery + Finisher	
	Estimate^a^ (SE)	p-value	Estimate (SE)	p-value	Estimate (SE)	p-value
KLH^b^-G^f^	−0.009 (0.039)	0.811	−0.101 (0.032)	0.002	−0.075 (0.027)	0.005
LPS^c^-G	−0.020 (0.034)	0.564	−0.080 (0.028)	0.005	−0.062 (0.024)	0.009
LTA^d^-G	−0.069 (0.048)	0.148	−0.039 (0.040)	0.921	−0.026 (0.033)	0.435
PDG^e^-G	−0.018 (0.023)	0.455	−0.062 (0.019)	0.001	−0.045 (0.016)	0.005
Ave-G^h^	−0.031 (0.028)	0.265	−0.063 (0.022)	0.006	−0.053 (0.020)	0.006
KLH-M^g^	−0.013 (0.045)	0.773	−0.038 (0.038)	0.307	−0.034 (0.030)	0.258
LPS-M	−0.027 (0.045)	0.541	−0.035 (0.037)	0.340	−0.042 (0.030)	0.160
LTA-M	−0.051 (0.070)	0.460	−0.067 (0.058)	0.250	−0.021 (0.047)	0.657
PDG-M	−0.030 (0.041)	0.457	−0.044 (0.034)	0.194	−0.045 (0.028)	0.105
Ave-M^i^	−0.060 (0.041)	0.884	−0.045 (0.034)	0.177	−0.036 (0.28)	0.191
IgG-T^j^	−0.042 (0.038)	0.266	−0.091 (0.032)	0.004	−0.069 (0.026)	0.008

^a^Difference in log_e_ (sample/positive ratio) of pigs that died minus that of pigs that survived

^b^KLH = keyhole limpet hemocyanin

^c^LPS = lipopolysaccharide

^d^LTA = lipoteichoic acid

^e^PDG = peptidoglycan

^f^G = isotype IgG

^g^M = isotype IgM

^h^Ave-G = the average of log_e_NAb IgG across the four antigens

^i^Ave-M = the average of log_e_NAb IgM across the four antigens

^j^IgGT = the S/P ratio for total IgG level in plasma

**Fig. 3:**
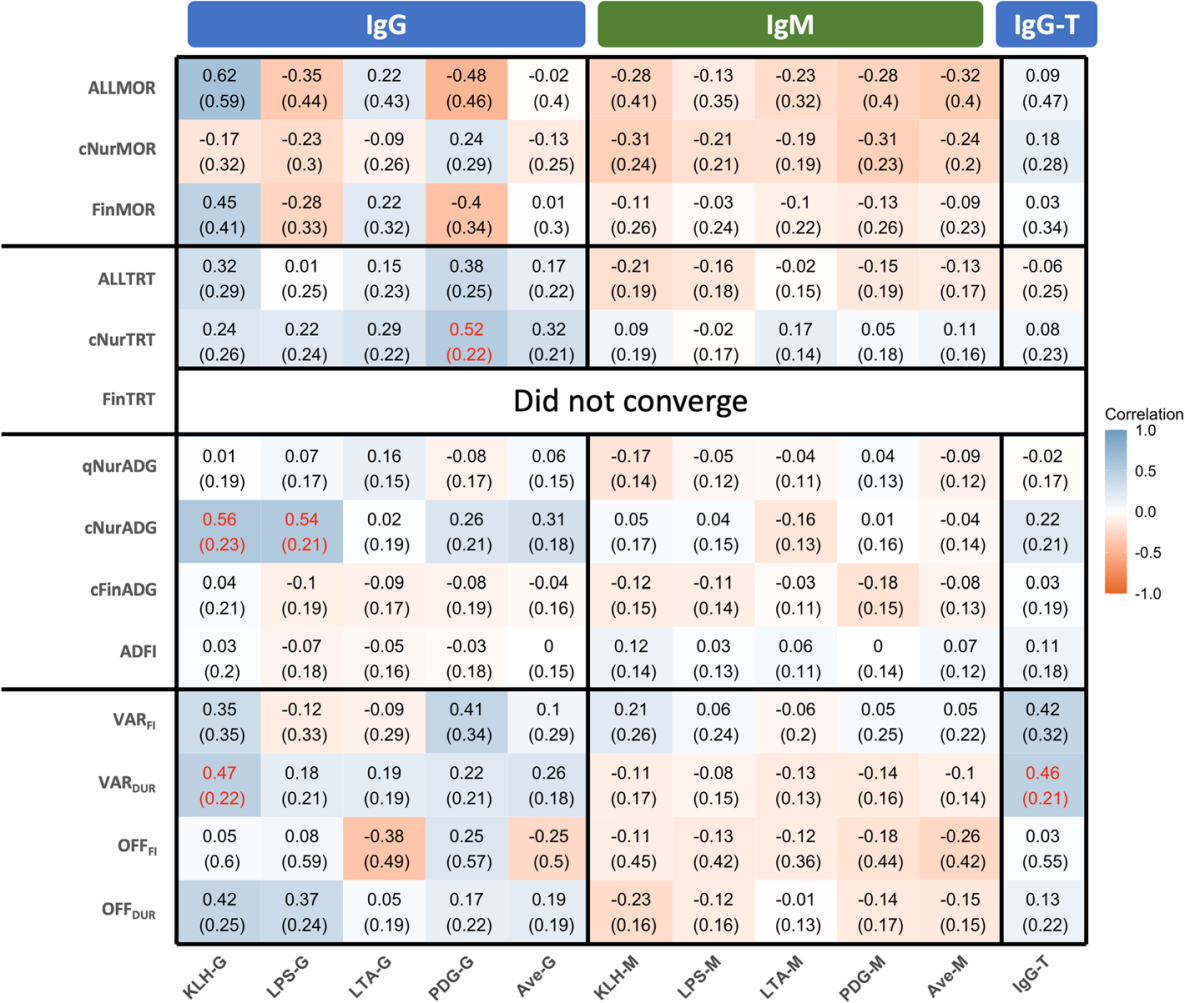
Estimates of genetic correlations of log_e_-transformed sample/positive ratios IgG and IgM natural antibodies against four antigens and for total IgG in blood of young healthy piglets with their subsequent performance and resilience phenotypes during a natural polymicrobial disease challenge. Red value means the correlations is significant (p-value is less than 0.05 based on z test). KLH = keyhole limpet hemocyanin. LPS = lipopolysaccharide. LTA = lipoteichoic acid. PDG = peptidoglycan. Ave-G = the average NAb IgG isotype binding to the four antigens. Ave-M = the average NAb IgM isotype binding to the four antigens. IgG-T = total IgG level in plasma (unit is mg/ml). G = isotype IgG binding to the four antigens. M = isotype IgM binding to the four antigens. ALLMOR = 1 for pigs that that died prior to slaughter; = 0 for pigs that survived to slaughter. cNurMOR = mortality for challenge nursery period. FinMOR = mortality for challenge finishing period. ALLTRT = treat rate adjust to 180 days. qNurADG = average daily gain for quarantine nursery period. cNurADG = average daily gain for challenge nursery period. cFinADG = average daily gain for challenge finishing period. ADFI = average daily feed intake. VAR_FI_ = the root mean square error from the within individual regression of feed intake on age. VAR_DUR_ = the root mean square error from the within individual regression of feed intake duration on age. OFF_FI_ = the proportion of negative residuals for 5% quantile regression of feed intake on age across all pigs. OFF_DUR_ = the proportion of negative residuals for 5% quantile regression of feed intake duration on age across all pigs

Figure 3 shows estimates of genetic correlations of antibody levels with performance and resilience traits. Genetic correlations of NAb and IgG-T with resilience traits were stronger than the corresponding phenotypic correlations, but they also had much larger standard errors. Estimates of the genetic correlation of antibody levels with ALLMOR and ALLTRT were not significant, except for the correlation of PDG-G with cNurTRT (0.52 + 0.22). Estimates of genetic correlations of IgM NAb with ALLMOR and ALLTRT tended to be negative, while IgG NAb tended to be positively correlated with ALLTRT and cNurTRT. The direction of the correlations of cNurADG with NAb and IgG-T was not consistent. Growth rate in the challenge nursery had significant positive genetic correlations with KLH-G NAb (0.56 ± 0.23) and LPS-G (0.54 ± 0.21). VAR_DUR_ was positively correlated with IgG NAb (0.18 to 0.47), of which the genetic correlation with KLH-G was significant (0.47 ± 0.22). VAR_DUR_ also had a significant positive genetic correlation with IgG-T (0.46 ± 0.21). However, none of the genetic correlations of VAR_DUR_ with IgM NAb were significant (− 0.14 to − 0.08). The direction of the genetic correlations with NAb and IgG-T were similar for OFF_DUR_ as for VAR_DUR_, but none of these estimates were significantly different from zero.

### Genome-wide association analyses

**Fig. 4:**
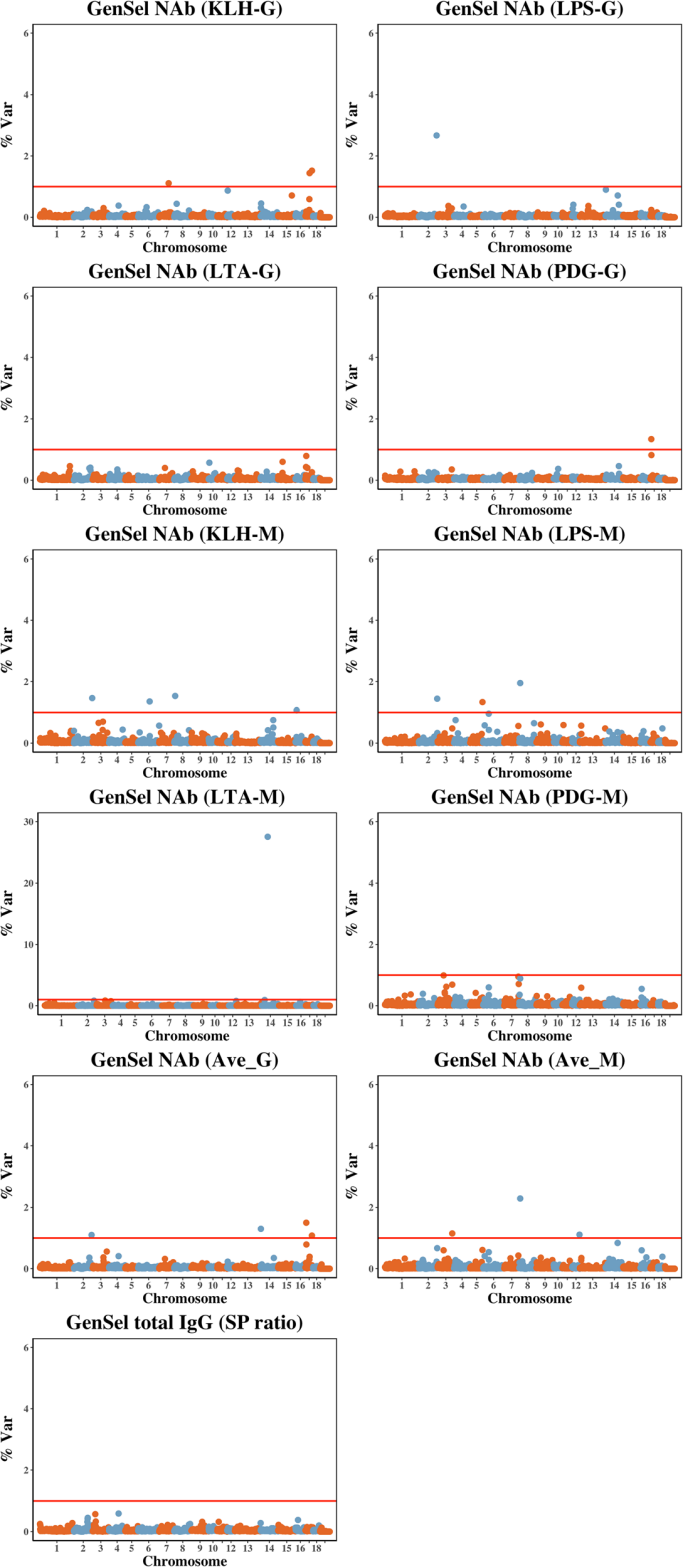
Manhattan plots for the percent of genetic variance of log_e_-transformed sample/positive ratios for IgG and IgM natural antibodies against four antigens and for total IgG in blood of young healthy piglets that is explained by 1-Mb non-overlapping windows. Red lines indicate the percent of genetic variance is 1

Manhattan plots for NAb and IgG-T levels are shown in Fig. 4 . Three, one, and one 1-Mb regions were associated with KLH-G, LPS-G, and PDG-G, respectively, based on explaining more than 1% of genetic variance (Table 4). Four 1-Mb regions were associated with average NAb-G, two of which were also found for KLH-G and PDG-G. Four, three, and one 1-Mb regions were associated with KLH-M, LPS-M, and LTA-M, respectively, while three 1-Mb regions were associated with the average IgM NAb, one of which, located on chromosome 8, was also identified for LPS-M. There were no significant genome regions that were associated with IgG-T levels.

**Table 4:** One-Mb genome regions associated with log_e_-transformed sample/positive ratio for IgG and IgM natural antibodies against four antigens and for total IgG in blood of young healthy piglets based on the % of genetic variance explained (%Var)

Antibody	Chromosome	Mb Window	%Var
**KLH-G**^**a**^	17	54	1.5
	17	34	1.4
	7	76	1.1
**LPS-G**^**b**^	2	148	2.7
**PDG-G**^**c**^	17	7	1.3
**Ave-G**^**d**^	17	7	1.5
	14	2	1.3
	2	143	1.1
	17	53	1.1
**KLH-M**^**e**^	6	7	1.5
	2	148	1.5
	6	93	1.4
	16	9	1.1
**LPS-M**^**f**^	8	7	2.0
	2	148	1.4
	5	99	1.3
**LTA-M**^**g**^	14	48	27.5
**Ave-M**^**h**^	8	7	2.3
	3	118	1.2
	12	59	1.1

^a^KLH-G = NAb isotype IgG binding to keyhole limpet hemocyanin

^b^LPS-G = NAb isotype IgG binding to lipopolysaccharide

^c^PDG-G = NAb isotype IgG binding to peptidoglycan

^d^Ave-G = the average NAb IgG isotype binding to the four antigens

^e^KLH-M = NAb isotype IgM binding to keyhole limpet hemocyanin

^f^LPS-M = NAb isotype IgM binding to lipopolysaccharide

^g^LTA-M = NAb isotype IgM binding to lipoteichoic acid

^h^Ave-M = the average NAb IgM isotype binding to the four antigens

Identified associated genomic regions were screened to identify positional candidate genes (Supplementary Tables 1 and 2). In total, 253 and 232 positional candidate genes were found for IgG and IgM NAb, respectively. The positional candidate genes did not represent significant functional annotation clusters. However, of the 253 positional candidate genes for IgG NAb, 13 genes are annotated to be involved in immune processes (*CD14, ANKHD1, DEFB116, DEFB123, DEFB124, DEFB125, DEFB128, DEFB129, NFIL3, SYK, TRDC, IRF9, IL25*), while 9 are involved in innate immune response (*CD14, ANKHD1, DEFB116, DEFB123, DEFB124, DEFB125, DEFB128, DEFB129, SYK*). Of the 232 positional candidate genes associated with IgM NAb, five are annotated to be involved in immune response (*LIF, MIF, OSM, SUSD2, CSF3R*), while one (*MIF*) is involved in innate immune response. Four candidate genes associated with immune response (*LIF, MIF, OSM, SUSD2*) were located in the genomic region for LTA-M on chromosome 14 around 48 Mb, which explained 27.5% of the genetic variance.

## Discussion

This study analyzed data from blood samples collected on young, healthy piglets that were subsequenty entered into a natural polymicrobial disease challenge to: (1) estimate genetic parameters of antibody levels (NAb and total IgG) in plasma of young, healthy pigs; (2) evaluate the potential of these NAb and IgG levels as indicator traits to select for disease resilience; and (3) conduct GWAS analysis for these antibody levels. To the best of our knowledge, this represents the first report on the genetic basis of NAb in the blood of pigs and their relationship with disease resilience.

NAb levels of two isotypes (IgG and IgM) associated with four important PAMPs (KLH, LPA, LTA and PDG) were quantified. NAb are non-inducible, meaning that their levels do not increase following exposure to key epitopes. Piglets are, however, exposed to multiple PAMPs during early life, and potentially in utero and during birthing. Three of the antigens used in this study (LPS, LTA, PDG) have PAMP epitopes that are common to Gram-negative and Gram-positive bacteria. However, KLH has PAMP epitopes derived from the inedible mollusc, *Megathura crenulate*, which commercial piglets are very unlikely to have been exposed to, although KLH NAb could cross-react with other antigens that have a similar structure. In spite of this apparent lack of specificity, which is common to IgM and the earliest IgG antibodies produced, the fact that these antigens are readily available and their respective antibodies can be easily measured in blood, makes them attractive targets for as indicator traits for disease resilience.

### Genetic parameters of NAb

Genetic parameters for NAb in blood have been studied in several other livestock species but not in pigs. In poultry, Siwek et al. [29] estimated the heritability for NAb binding LPS and LTA in blood from different chicken lines and at different ages to range from 0.09 to 0.23 and from 0.03 to 0.42, respectively; Wijga et al. [25] estimated a heritability of 0.23 for the titer of NAb binding rabbit red blood cells in chickens. Thompson-Crispi et al. [24] estimated the heritability of IgM and IgG NAb binding KLH to be 0.32 and 0.18, respectively. Klerk et al. [22] found that the heritability of NAb was higher for IgM (0.25) than for the IgG isotype (0.15). Ploegaert et al. [14] estimated intra-herd heritabilities for NAb titers in milk of Dutch Holstein-Friesian cows to range from 0.10 to 0.53. In our study, estimates of the heritability of NAb titers in young healthy pigs ranged from 0.12 to 0.53, which is within the published range of estimates from previous studies [14, 24, 30]. In our study, heritability estimates were low for IgG NAb but moderately high for IgM NAb. Estimates of the proportion of variance explained by maternal effects for IgG and IgM NAb showed the opposite trend, being moderately high for IgG NAb and low for IgM NAb.

Natural antibodies consist of three isotypes: IgM, IgG and IgA. A previous study found that immunoglobulins IgG, IgM and IgA can transfer to lacteal secretions in the parturient sow and be absorbed by neonatal piglets [31]. In humans, IgG has been found to be the only isotype that has the ability to significantly cross the placental barrier to provide immunity to the fetus [32]. Previous studies in cattle have also shown that colostrum contains IgG NAb [33]. Because IgG transfers between the dam and fetus in pigs, part of the IgG NAb measured in piglet plasma likely originated from the colostrum from the dam, which explains the high litter effects for NAb IgG and IgG-T. Porter [31] found that more than 60% of whey protein in milk of sows was immunoglobulin, with the IgG isotype accounting for the majority (79.7%), while the IgM isotype content was only 6.27%. The contents IgG and IgM in colostrum are, however, different, which might lead piglets to get more IgG and less IgM from colostrum, explaining the higher litter effects for IgG NAb than for IgM NAb.

Ploegaert et al. [14] showed that the genetic correlation between titers of total NAb (IgG, IgM, and IgA) binding the same four antigens as used in our study ranged from 0.45 to 0.99 in milk of Dutch Holstein-Friesian cows and the titers of IgG NAb binding KLH in milk was highly correlated with the corresponding titer in serum (0.70). The estimates of the genetic correlations between NAb (IgG and IgM) binding the four antigens obtained in our study (0.41 to 0.99) are in agreement with these literature estimates. In dairy cows, Thompson-Crispi et al. [24] estimated a negative genetic correlation (− 0.41) between IgG NAb and IgM NAb titers in milk, which contrasts with the moderate positive estimates in our study.

Our results show that a given NAb isotype binding different antigens has a common genetic basis and selection of any of the four antigens is expected to result in correlated responses in the same direction for antibodies to the other PAMPs. Hence, there may not be a need to measure NAb levels for all four antigens in genetic selection programs. The KLH NAb were strongly correlated with NAb to the other three antigens, and therefore, may be a reliable measure of NAb, given its unique nature.

Few studies have assessed the genetic and phenotypic relationships of NAb with total IgG titers in blood. In the current study, total IgG had lower genetic and phenotypic correlations with IgM NAb than with IgG NAb, except for LTA binding IgM NAb. A recent study by Berghof et al. [17] in chickens estimated the phenotypic and genetic correlations of KLH binding IgG NAb with total IgG to be 0.08 and − 0.61, respectively, but with a high standard error for the latter (0.55). Our estimate of the genetic correlation of IgG-T with KLH-G was strong and positive (0.81) and the phenotypic correlation was also positive (0.51). Possible reasons for the differences between these estimates may be the species studied, differences in age and prior exposure to pathogens, and the large standard errors of the estimates.

### Relationships of antibody levels with resilience

NAb levels prior to challenge had weak phenotypic correlations with subsequent disease resilience and performance traits after challenge, although some were significant and in the expected direction. For example, some NAb levels were negatively correlated with mortality, the number of parenteral treatments, and day-to-day variation in duration at the feeder (VAR_DUR_), and positively correlated with growth rate. Consistent with these correlations, we also found that pigs that died had significantly higher NAb for some antigens and isotypes than pigs that survived. Estimates were, however, too small to serve as reliable phenotypic predictors of resilience. Star et al. [27] reported that the levels of NAb binding KLH and LPS were phenotypically positively correlated with the probability that chickens survive during the laying period. Sun et al. [34, 35] also revealed that IgG and IgM NAb levels in blood, especially IgM binding KLH, were phenotypically positively correlated with survival rate to around 20 weeks of age in both purebred and crossbred laying hens.

In the present study, several NAb traits had moderately high estimates of genetic correlations with some disease resilience and performance traits following challenge. All IgM NAb levels had negative genetic correlation estimates with mortality. While in the expected direction, none of these estimates were significantly different from zero. Estimates of the genetic correlation of IgG NAb and IgG-T with resilience and performance traits following challenge did not have a consistent direction and had high standard errors, likely because of the lower heritability for IgG NAb compared to IgM NAb. All IgG NAb traits had positive genetic correlation estimates with the number of parenteral treatments, with the estimate of PDG-G with the number of treatments in the challenge nursery being significantly different from zero. The estimate of the genetic correlation of IgM NAb with the number of treatments was not significantly different from zero. The direction of estimates of genetic correlations of NAb levels with growth rate were also mixed, but the significant correlations were positive (i.e. of growth rate in the challenge nursery with KLH-G and LPS-G), as expected.

Putz et al. [6] showed that smaller day-to-day variation in feed intake (VAR_FI_) or duration (VAR_DUR_) and the proportion of off-feed days based on negative residuals for 5% quantile regression of feed intake (OFF_FI_) or duration (OFF_DUR_) on age were genetically correlated with improved disease resilience and performance under disease challenge in the NDCM. In the present study, most estimates of the genetic correlation of these traits with IgG NAb and IgG-T were positive, opposite to expectations. The estimate of the genetic correlations of VAR_DUR_ with KLH-G was significant and similar to the unexpected positive correlation of KLH-G with growth rate in the challenge nursery, as well as the estimate for VAR_DUR_ with total IgG.

In general, compared with IgG NAb and total IgG, IgM NAb had a relatively consistent genetic correlation estimates with resilience and performance traits under challenge, especially with mortality, and their direction was consistent with expectations.

### Genomic regions associated with antibody levels

Several genomic regions that were significantly associated with antibody levels were identified in the GWAS and numerous positional candidate genes were identified for these regions (Table 4). None of the positional candidate genes identified by the GWAS analysis in this study have been reported in previous studies on NAb, except the CD14 gene on chromosome 2, which will be discussed in the following.

Functional annotation analysis of these positional candidate genes for IgG NAb did not identify annotated functional clusters. However, 13 genes related to the immune process were identified. In poultry, Berghof et al. [17] identified a significant genomic region associated with KLH-M and total IgM concentration that contains the TLR1A gene. Although the TLR gene was not in the significant genomic regions identified in our study, the significant regions we identified on chromosome 2 around 143 Mb for average IgG NAb and around 148 Mb for LPS-G, KLH-M and LPS-M are close to the CD14 gene. This gene has been reported to encode proteins involved in inflammatory and innate immune responses and, together with TLR-4 and MD-2, acts as a common receptor for bacterial LPS [36]. The TLR signaling pathway is critical for innate immunity and provides the first line of defense against antigens.

The 148 Mb region of chromosome 2 also contains the ankyrin repeat and KH domain-containing protein 1 (ANKHD1) gene, which is involved in the immune process. Miles et al. [37] found that a novel splice variant of ANKHD1 may play a role in the apoptosis and cell survival pathway. Machado-Neto et al. [38] found that the ANKHD1 gene is involved in the Hippo signaling pathway and can promote cell growth and cell cycle progression by upregulating Cyclin A. In an acute leukemia human study, Traina et al. [39] found that the ANKHD1 gene may be related with the abnormal phenotype of leukemia cells as a scaffold protein. It was also found that the ANKHD1 gene can reduce cell growth and delay cell cycle progression in the S phase.

Three immune genes were detected in the significant genomic region for KLH-G (at 76 Mb on chromosome 7), i.e. T cell receptor delta chain C region (*TRDC*), interferon regulatory factor 9 (*LRD9*), and interleukin 25 (*IL25*). *TRDC* participates in antigen recognition [40], especially for a variety of self and foreign non-peptide antigens that are frequently expressed at the epithelial boundaries between the host and external environment. Once the antigen is recognized, a rapid, innate-like immune response is produced to participate in pathogen clearance and tissue repair [41, 42]. *LRD9* is a transcription factor that can mediate signaling by type I interferons. After type I interferons bind to cell surface receptors, Jak kinases (*TYK2* and *JAK1*) are activated, which leads to tyrosine phosphorylation of signal transducer and activator of transcription 1 (*STAT1*) and signal transducer and activator of transcription 2 (*STAT2*). *IRF9*/interferon stimulates transcription factor 3 gamma (*ISGF3G*) to associate with the phosphorylated *STAT1*:*STAT2* dimer to form a complex called the ISGF3 transcription factor, which can enter the nucleus. Interferon stimulates transcription factor 3 (*ISGF3*) binds to the interferon stimulated response element (*ISRE*) to activate the transcription of interferon stimulated genes, which drives the cell into an antiviral state [43]. *IL25* is involved in cytokine binding and inflammatory response. and encodes cytokine proteins, which can induce NF-kappaB activation and stimulate the production of interleukin 8 [44]. Another important function for *IL25* is promoting the development of a Th2 immune response, which can protect against bowel infection by helminths [45, 46].

A series of beta defensin genes are located in the significant genomic region for IgG at 35 Mb on chromosome 17. Beta-defensin belong to the mammalian defensin family and are involved in the first line of defense of innate immunity [47]. Defensin helps to protect against a variety of microbes, including Gram-positive and Gram-negative bacteria, fungi, yeast and enveloped viruses [48]. Defensins may be involved in linking innate and adaptive immune response, and have been found to act as signal molecules of the immune system and a chemokine for T lymphocytes and immature dendritic cells [49].

The NFIL3 gene is located in a significant genomic region at 2 Mb on chromosome 14 associated with IgG NAb. The NFIL3 gene regulates the transcription of interleukin-3 (IL3), which controls IgE class switching [50]. Kashiwada et al. [51] found that NFIL3 was the key regulator of type 2 helper cells, which are important for the development of allergic immune responses. The spleen tyrosine kinase (SYK) gene is located in the same genomic region (at 2 Mb on chromosome 14) that was found to be associated with IgG NAb. This gene is involved in multiple biological processes, including innate immune recognition, cell adhesion, platelet activation, and vascular development [52].

Although functional annotation analysis did not identify gene clusters that were associated with the identified positional candidate genes for IgM NAb, four genes associated with immune response were identified in the genome region chromosome 14 around 48 Mb. These include the leukemia inhibitory factor (LIF), macrophage migration inhibitory factor (MIF), oncostatin M (OSM), and sushi domain containing 2 (SUSD2) genes. LIF is a multifunctional cytokine that mediates neuronal, hepatic, endocrine, inflammatory, and immune responses in autocrine and paracrine manners [53]. MIF is involved in the host antimicrobial alarm system and stress response, which can promote pro-inflammatory functions [54]. The MIF proteins are released by stimulated white blood cells and produce an acute immune response by binding to CD74 on immune cells such as macrophages, lymphocytes, dendritic cells, and endothelial cells [55]. The OSM gene has been reported to be an inflammatory mediator, similar to cytokines, but its exact effect on the immune system is unknown [56]. The SUSD2 gene is involved in the invasiveness of breast cancer cells and tumor evasion [57]. The *CSF3R* gene, which is associated with cytokine binding and cytokine receptor activity processes, is located in the genome region around 92 Mb on chromosome 6. This gene can encode the receptor protein for colony stimulating factor 3, which controls the production, differentiation, and function of granulocytes. After ligand binding the CSF3R, the receptor undergoes a conformational change, which can active the downstream pathways including JAK/STAT, PI3K/AKT, and MAPK/ERK [58].

### Comparison with other innate immunity and immunocompetence traits in pigs

Several previous studies have estimated genetic parameters of immune traits in blood and provided a genetic framework for potential immune options in pigs [59–61]. Clapperton et al. [59, 60] showed that several traits measured in blood (white blood cells and peripheral blood mononuclear leucocyte subsets) that are related to the innate and adaptive immunity, are heritable and genetically negatively correlated with growth performance under different health status conditions. This suggests that these immune traits could be the potential genetic predictors of performance under different health conditions, which agrees to some extent with our results for NAb. In a review on piglet survival, Heuß et al. [62] argued that it is necessary to investigate the relationship of immune parameters with the robustness, survival, and performance. Our study offers insights into the genetic relationships between immune traits measured in young healthy pigs and disease resilience and production traits under disease, including survival and performance.

### Limitations of natural polymicrobial disease challenge

The disease challenge model used here was designed to mimic a severe disease challenge on commercial farms with multiple pathogens, to maximize the expression of disease resilience. Because of seasonal effects, necessary veterinary interventions, and the dynamic nature of natural transmission of pathogens in a barn, pathogen exposure was not constant from batch to batch, similar to a commercial situation, where pathogen profiles varies widely between farms and over time within a farm. However, by evaluating pigs from multiple batches, the relationships identified in this study are expected to be robust to the specific level and nature of pathogen exposure. Also, because of the polymicrobial dynamic disease pressure, there was little value in determining the cause of each death.

## Conclusions

Overall, the results of this study suggest that levels of NAb and total IgG in plasma of young healthy pigs have potential as indicators of disease resilience, especially the IgM isotype NAb due to its higher heritability and lower litter effects. Of the four PAMPs binding IgM isotype NAb, KLH is the most promising because piglets may be exposed to the other three PAMPs even in a biosecure environment. Further research is, however, required to validate the identified relationships of these NAb with disease resistance and resilience.

## Methods

The protocol of this project was approved by the Animal Protection Committee of the Centre de recherche en sciences animales de Deschambault (15PO283) and the Animal Care and Use Committee of the University of Alberta (AUP00002227), and was based on the Canadian Council on Animal Care guidelines (CCAC; https://www.ccac.ca/en/certification/about-certification). Comprehensive supervision of animal care was provided by the Centre de développement du porc du Québec (CDPQ) and the herd and project veterinarians. If needed, pigs in the natural disease challenge were humanely euthanized. Following CCAC guidelines, electrocution was used in the nursery and cranial captive bolt during the finisher period. Pigs that reach slaughter weights were stunned by electrocution at a commercial slaughter facility, followed by exsanguination, using standard approved industry protocols.

### Study design

Putz et al. [6] provided a detailed description of the natural disease challenge model (NDCM) for wean-to-finish pigs that generated the data used in this study. Briefly, the NDCM consisted of three phases: (1) a 19 day quarantine nursery period, starting at ~ 21 days of age; (2) a 28 day challenge nursery period, starting at ~ 40 days of age; and (3) a challenge finisher period from ~ 70 days of age to slaughter at ~ 26 weeks of age. Every 3 weeks, a batch of 60 to 75 weaned, Large White by Landrace crossbred barrows from a high-health multiplier farm in Canada from one of the seven members of PigGen Canada (http://piggencanada.org/) entered the quarantine nursery, as described by Putz et al. [6]. Seven batches in rotation (one batch per company) were considered one cycle. For cycle 1, the quarantine nursery was an isolated room within the challenge facility with operational biosecurity procedures designed to minimize pathogen transmission from the challenge areas, while for cycles 2 to 7, the quarantine nursery was a building located on a separate, biosecure site about 1 km south of the challenge facility. This study utilized antibody levels measured on blood samples from 1799 pigs in the first four cycles, and performance and resilience data from 3205 pigs from cycles 1 to 7.

The natural polymicrobial disease challenge was established during the first 4 batches of cycle 1 by introducing seeder pigs from neighboring commercial farms into the challenge barn (challenge nursery and finisher phases) that were naturally infected with porcine reproductive and respiratory syndrome virus, porcine circovirus type 2, *Mycoplasma hyopneumoniae*, *Actinobacillus pleuropneumoniae*, influenza A virus of swine, and various opportunistic bacterial pathogens including *Streptococcus suis*, *Haemophilus parasuis*, pathogenic *E. coli*, *Salmonella* spp., *Lawsonia intracellularis*, and *Brachyspira* spp.. Once established, pathogen circulation was maintained by direct and indirect contact with pigs from older batches in a continuous flow system, with careful veterinary oversight and group and individual antibiotic treatments, as necessary, to maintain a balance between disease pressure and animal welfare.

### Antibody level quantification

Blood samples were collected on all pigs in a batch via jugular vena puncture into plain vaccutainers at ~ 26 days of age, 5 days after entry into the quarantine nursery. Plasma was obtained by centrifugation (20 min at 750 × g) and frozen at − 20 °C until analysis. NAb levels were quantified using an indirect, two-step enzyme linked immunosorbent assay (ELISA) adapted from Van Knegsel et al. [10] for use in plasma of pigs. In short, NAb antigens: KLH (keyhole limpet hemocyanin from *Megathura crenulata*; MP Biomedicals, Irvine, CA., USA), LPS (lipopolysaccharides from *Escherichia coli* 055:B5; Sigma, St. Louis, MO., USA), LTA (lipotechoic acid from *Staphylococcus aureus*; Sigma, USA), and PDG (peptidoglycan from *Staphylococcus aureus*; Sigma) were prepared as 1 mg/mL stock solutions in 0.05 M carbonate buffer, pH 9.6 (Sigma), then aliquoted and stored at − 80 °C. Immulon 4 HBX ELISA plates (Fisher, Ottawa, ON., Canada) were coated with 100 μL of 1 μg/mL KLH, 4 μg/mL LPS, 5 μg/mL LTA, or 2 μg/mL PDG in carbonate buffer and incubated overnight at 4 °C. After warming 2 h at 22 °C, plates were washed five times in a Bio-Rad Bio-Plex Pro II Wash Station with 50 mM Tris buffered saline, pH 8.0/0.05% Tween 20 (Sigma). Wells were then blocked with 200 μL of 1% BSA in 50 mM Tris, pH 8.0 (Sigma) for 30 min at 22 °C. Plates were washed as above before adding 100 μL of plasma diluted 1:40 in 1% BSA in Tris, pH 8.0/0.05% Tween 20 (sample/conjugate diluent) in duplicate wells. An appropriately diluted control was added in duplicate to each plate, consisting of a known positive pooled plasma sample specific to the antigen and antibody isotype that was aliquoted and stored at − 80 °C. Each plate also contained two wells with sample/conjugate diluent alone as blanks. After 1 h at 22 °C, plates were rewashed as described above. Either 100 μL of goat anti-pig IgG-Fc Fragment HRP conjugated antibody (Bethyl Laboratories, Montgomery, TX., USA) diluted 1:40,000 in sample/conjugate diluent or 100 μL of goat anti-pig IgM HRP conjugated antibody (Bethyl) diluted 1:10,000 in sample/conjugate diluent was added for 1 h at 22 °C. Plates were washed and 100 μL/well of TMB One Component HRP Microwell Substrate (Bethyl) was added per well. Color development in the dark at 22 °C was monitored at 650 nm in a Molecular Devices Vmax Microplate Reader using SoftMaxPro software v5.4 (Sunnyvale, CA, USA). Stop Solution (100 μl; 0.18 M H_2_SO_4_; Bethyl) was added when plate control wells read about 0.7, or at 30 min, whichever was sooner. Final results were obtained at 450 nm and expressed as ratios of sample to plate control (S/P; Formula 1), after values obtained from the blank well on that plate were deducted:

1 $$ \mathrm{S}/\mathrm{P}=\frac{OD_{Sample}-{OD}_{Negative\_ Control}}{OD_{Positive\_ Control}-{OD}_{Negative\_ Control}} $$

Porcine total IgG was quantified by ELISA using a commercial kit (Cat E100–104, Bethyl Labs Inc., Montgomery, TX). Briefly, 96 well plates were coated with goat anti-pig IgG-Fc coating antibody. Blocking solution, test sera (diluted 1:40,000), HRP conjugated goat anti-pig IgG-Fc detection antibody (diluted 1:100,000) and TMB substrate solution were added to each well in a stepwise manner following the appropriate incubation at room temperature and plate washes required between each solution. Plates were developed in the dark for 15 min before stop solution was added. Absorbance was read at 450 nm. Included on each plate were standards that were comprised of reference sera (provided with the kit) diluted 10,000 to 0 ng/ml. Test sera and standards were run in duplicate. Duplicates with CV greater than ~ 10% were re-run.

### Performance and resilience phenotypes

Performance and resilience phenotypes were collected in the challenge nursery and finisher, as described by Putz et al. [6]: mortality (1 for pigs that that died prior to slaughter; 0 for pigs that survived to slaughter) for the whole challenge period (ALLMOR), in the challenge nursery (cNurMOR), and in the finisher (FinMOR); the number of individual parenteral antibiotic treatments during the entire challenge period, adjusted to 180 days (nTrtPer180), during the challenge nursery period, adjusted to 27 days (cNurTRT), and during the finishing period (FinTRT), adjusted to 100 days; average daily gain (ADG) in the quarantine nursery (qNurADG), in the challenge nursery (cNurADG), and in the finisher (cFinADG) periods, based on loess regression of individual weights recorded every 3 weeks; average daily feed intake in the finisher (ADFI); the root mean square error of the regression of daily feed intake (VAR_FI_) or daily feed intake duration (VAR_DUR_) in the finisher on age, and the proportion of negative residuals from 5% quantile regression of daily feed intake (OFF_FI_) or daily feed intake duration (OFF_DUR_) across all animals. Pigs that died during a given phase (nursery or finisher) received a missing value for phenotypes recorded in that phase, except for mortality.

### Genotyping and quality control

Genotypes were obtained on all pigs in the 7 cycles, using a 650 k Affymetrix Axiom Porcine Genotyping Array by Delta Genomics (Edmonton AB, Canada). In total, the chip contained 658,692 single nucleotide polymorphisms (SNPs). Delta Genomics used default settings of Axiom® Analysis Suite (quality control thresholds: SNP call rate ≥ 97%; number of minor alleles observed ≥ 2) to process the raw SNP data separately for each cycle. After quality control, 435,172 SNPs on 3205 pigs remained for analysis. Additional details of quality control methods are in Putz et al. [6].

### Statistical analyses

#### Genomic relationship matrix

The PreGSf90 software of BLUPF90 [63] was used to process the SNP genotype data and created the additive genomic relationship matrix. The additive genomic matrix was estimated separately for each company and then combined into one large matrix. Genetic relationships between companies were set to be zero in order to focus on pooled within-company variances.

#### Genetic parameters of antibody traits

To improve normality of distributions, all antibody values were transformed using the natural logarithm function. The following single-trait, mixed linear animal model was used to estimate the variance components and genetic parameters for each antibody trait:

2 $$ \boldsymbol{y}=\boldsymbol{Xb}+\boldsymbol{Sl}+\boldsymbol{Vu}+\boldsymbol{e} $$

where ***y*** is a vector of the log of antibody levels; ***X*** is the design matrix associated with ***b*,** which is a vector of the fixed effect of batch and the covariate of age at entry into the quarantine nursery; ***S*** is an incidence matrix associated with ***l*,** which is a vector of random effects of litter and pen, assumed to be distributed *N* $\left(\mathbf{0},{\boldsymbol{I}\sigma}_l^2\right)$ and distributed *N* $\left(\mathbf{0},{\boldsymbol{I}\sigma}_p^2\right)$; ***V*** is an incidence matrix associated with ***u*,** which is a vector of random animal polygenic effects, assumed distributed N $\left(0,{\boldsymbol{G}\upsigma}_u^2\right)$, where ***G*** is the additive genomic relationship matrix based on SNP genotypes among animals within company (relationships between companies were set to zero); and ***e*** is a vector of random residual effects, assumed distributed *N* $\left(\mathbf{0},{\boldsymbol{I}\sigma}_e^2\right)$. Additive genetic variance (${\upsigma}_u^2$), litter variance (${\upsigma}_l^2$), pen variance (${\upsigma}_p^2$), and residual variance (${\upsigma}_e^2$) were estimated using restricted maximum likelihood using ASReml 4.0 [64]. Estimates of heritability, and proportion of variance due to litter effects were estimated as a proportion of phenotypic variance, which was the sum of ${\upsigma}_u^2$, ${\upsigma}_l^2$, and ${\upsigma}_e^2$. Phenotypic and genetic correlations among antibody level traits were estimated based on bivariate versions of model [2]. Standard errors of estimates were approximated as in ASReml 4.0 [52].

#### Association analysis of antibody levels with mortality

Association analyses of antibody levels with mortality during different time period were conducted using a model similar to model [2]. Mortality was fitted as a binary response variable in a mixed effect logistic regression model in R Studio, with the titer of each natural antibody was fitted as a covariate one at a time. The additive genomic relationship matrix was not fitted in this model. An ANOVA test was used to test whether the antibody level in blood had significant associations with mortality.

#### Correlations of antibody levels with performance and resilience traits

Phenotypic and genetic correlations of antibody traits with resilience traits were estimated using bi-variate models in ASReml 4.0, using model [2] for antibody traits and for the performance and resilience traits, except for pen effect corresponded to the pen relevant to the phase that the phenotype was recorded in. Phenotypic associations of antibody traits with mortality were also obtained as estimates of the effect of mortality on NAb based on model [2] with mortality (0/1 for pigs that survived/died prior to slaughter) added as a fixed effect.

#### Genome-wide association studies

Bayesian variable selection method Bayes-B [65], as implemented in GenSel version 4.90 [66], was used to identify associations of genomic regions with antibody levels. Each trait was analyzed separately. Because GenSel does not allow for inclusion of secondary random effects, antibody levels were pre-adjusted for estimates of litter and pen effects obtained from model [2]. The model used for GWAS analysis was:

3 $$ \boldsymbol{y}=\boldsymbol{x}b+{\sum}_{i=1}^k{\boldsymbol{z}}_i{\alpha}_i{\delta}_i+\boldsymbol{e} $$

where ***y*** is a vector of pre-adjusted antibody phenotypes; ***x*** is a vector of entry age of the piglets into the quarantine; *b* is a regression coefficient; ***z***_*i*_ is the vector of the additive genotype covariates for SNP *i*, coded as − 10, 0, 10 for genotypes AA, AB, and BB, respectively; *α*_*i*_ is the allele substitution effect of SNP *i*; *δ*_*i*_ is an indicator for whether the additive effect of SNP *i* was included in (*δ*_*i*_ = 1) or excluded from (*δ*_*i*_ = 0) from the model for a given iteration of the Markov chain Monte Carlo (MCMC) sampling method, with the prior probability (*π*) of *δ*_*i*_ = 0 set to 0.999; and ***e*** is the vector of residual errors assumed distributed ~ N $\left(0,{\boldsymbol{I}\sigma}_E^2\right)$. The MCMC was run for 41,000 iterations, with the first 1000 samples discarded as burn-in. Results were reported as the percent of genetic variance explained by non-overlapping 1-Mb windows of SNPs based on build 11.1 of the porcine genome (Ensemble, http://www.ensembl.org).

To identify potential genes associated with natural antibodies, 3 Mb genomic regions at and around a 1 Mb (1 Mb on either side) window that explained more than 1% of genetic variance were scanned for positional candidate genes against the swine genome (Sscrofa11.1, GCA_000003025.6, [67]) using the BioMart tool on the Ensembl website. Resolution of a GWAS depends in large part on the extent of linkage disequilibrium in the population analyzed. To evaluate the latter, we used the PLINK software [68] to compute linkage disequilibrium as a function of distance for SNPs on chromosome 7 (Supplemental figure 2). Results showed that the linkage disequilibrium was on average small when the distance was larger than 1 Mb. However, even at 1.5 Mb, there were pairs of SNPs that were in high linkage disequilibrium. This implies that a SNP that has a significant association with the trait, could be 1.5 Mb from the QTL. To account for this level of resolution, we evaluated the effects of 1 Mb windows, rather than of individual SNPs and choose a 3 Mb interval central on the 1 Mb window with substantial genetic variance to explore candidate genes. Identified positional candidate genes were submitted to the DAVID Bioinformatics Resources 6.8 program (https://david.ncifcrf.gov/) to identify their function and conduct functional annotation clustering. The genome background was set to be *Sus scrofa* and other parameters were set to default values. Benjamini adjusted *p*-values were used to determine whether a cluster was significant. A cluster was discarded if no term had a p-value less or equal to 0.05.

## Supplementary information

**Additional file 1: Figure S1.** Distribution of sample/positive ratios (S/P) for IgG and IgM natural antibodies and for total IgG in blood of young healthy piglets. **Figure S2.** The LD-decay in a r^2^ vs distance plot for chromosome 7. **Table S1.** The list of genes located in or around the significant genomic region windows associated with log_e_-transformed sample/positive ratios for IgG natural antibodies. **Table S2.** The list of potential genes around the significant genomic region windows associated with IgM NAb levels.

## Data Availability

The data analyzed in this study were obtained on commercial animals provided by members of the PigGen Canada consortium. Data can be made available upon reasonable request submitted to the corresponding author.

## Abbreviations

ADFI: Average daily feed intake
ANKHD1: Ankyrin repeat and KH domain-containing protein 1
cFinADG: average daily gain in the finishing period
cNurADG: average daily gain in the challenge nursery period
DEFB: Beta defensin
ELISA: Enzyme linked immunosorbent assay
GWAS: Genome-wide association studies
IgG-T: Total IgG
IL3: Transcription of interleukin-3
KLH: Keyhole limpet hemocyanin
LIF: Leukemia inhibitory factor
LPS: Lipopolysaccharide
LTA: Lipoteichoic acid
MIF: Macrophage migration inhibitory factor
NAb: Natural antibodies
NDCM: Natural disease challenge model
ALLTRT: The number of treatment rate adjusted to 180 days in the NCDM
cNurTRT: the number of treatment rate adjusted to 27 days during the challenge nursery in the NCDM
FinTRT: the number of treatment rate adjusted to 100 days during the finishing period in the NCDM
PAMP: Pathogen-associated molecular patterns
qNurADG: average daily gain in the quarantine nursery period
OFF_FI_: The proportion of negative residuals for 5% quantile regression of feed intake on age across all pigs
OFF_DUR_: The proportion of negative residuals for 5% quantile regression of feed intake duration on age across all pigs
OSM: Oncostatin M
PDG: Peptidoglycan
SNP: Single nucleotide polymorphism
SUSD2: Sushi domain containing 2
SYK: Spleen tyrosine kinase
TLR: Toll-like receptor
VAR_DUR_: The root mean square error of duration
VAR_FI_: The root mean square error of feed intake
