# Supplementary material for: The genetic basis of natural antibody titers of young healthy pigs and relationships with disease resilience
Source: BMC Genomics. 2020 Sep 22;21:648. doi: 10.1186/s12864-020-06994-0 (PMC7510148; doi:10.1186/s12864-020-06994-0)
Supplement: Supplementary file 1 — Additional file 1: Figure S1. Distribution of sample/positive ratios (S/P) for IgG and IgM natural antibodies and for total IgG in blood of young healthy piglets. Figure S2. The LD-decay in a r2 vs distance plot for chromosome 7. Table S1. The list of genes located in or around the significant genomic region windows associated with loge-transformed sample/positive ratios for IgG natural antibodies. Table S2. The list of potential genes around the significant genomic region windows associated with IgM NAb levels. [file 12864_2020_6994_MOESM1_ESM.docx]

Supplementary Figure 1. Distribution of sample/positive ratios (S/P) for IgG and IgM natural antibodies and for total IgG in blood of young healthy piglets


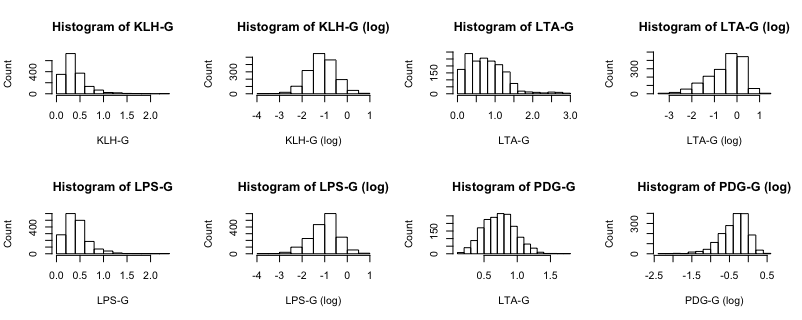


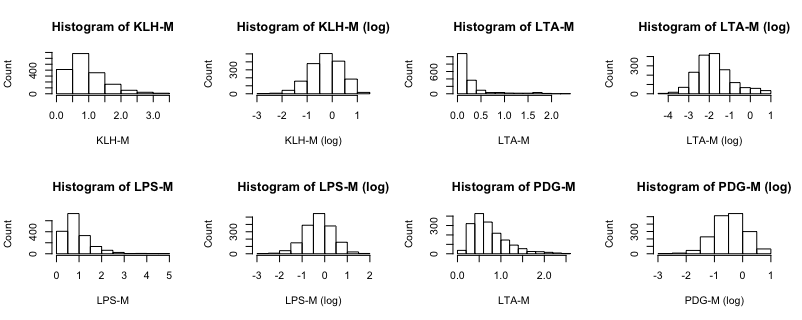


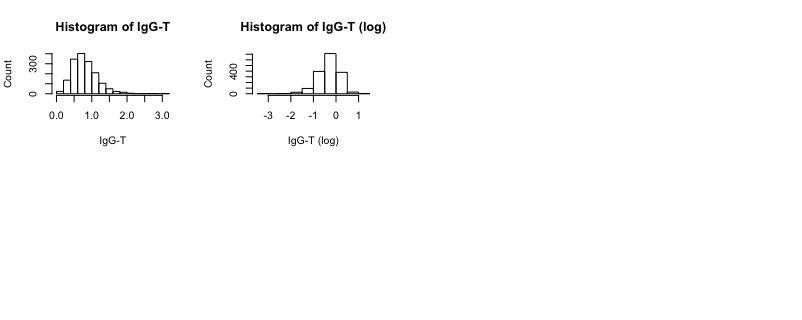


KLH = keyhole limpet hemocyanin IgG-T = total IgG level in plasma (unit is mg/ml)

LPS = lipopolysaccharide G = isotype IgG binding to the four antigens

LTA = lipoteichoic acid M = isotype IgM binding to the four antigens

PDG = peptidoglycan log = log_e_-transformed sample/positive ratios for antibodie

Supplementary Figure 2. The LD-decay in a r^2^ vs distance plot for chromosome 7.


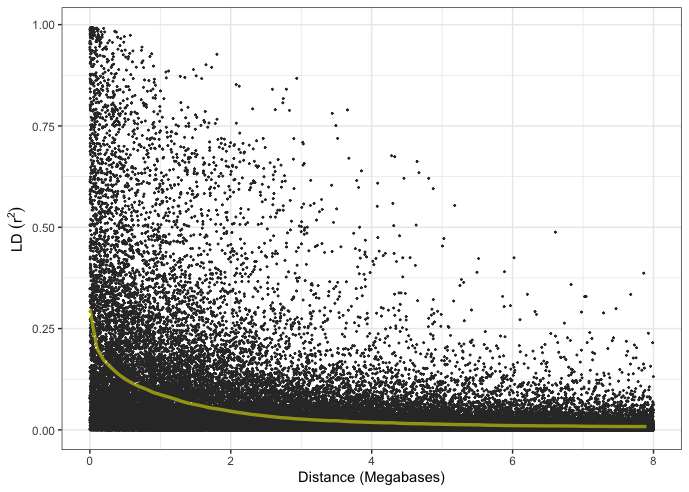


Supplementary Table 1. The list of genes located in or around the significant genomic region windows associated with log_e_-transformed sample/positive ratios for IgG natural antibodies.

| **Gene stable ID** | **Gene name** | **Chromosome** | **Gene start (bp)** | **Gene end (bp)** | **Phenotype associated with GWAS hit regions** |
| --- | --- | --- | --- | --- | --- |
| ENSSSCG00000014361 |  | 2 | 141878457 | 142010238 | **Ave-G** |
| ENSSSCG00000038493 | PFDN1 | 2 | 141991319 | 142077913 |  |
| ENSSSCG00000014362 | HBEGF | 2 | 142102458 | 142115196 |  |
| ENSSSCG00000014363 | SLC4A9 | 2 | 142129122 | 142140949 |  |
| ENSSSCG00000014364 | ANKHD1 | 2 | 142166314 | 142298097 |  |
| ENSSSCG00000019080 | RF00026 | 2 | 142279273 | 142279373 |  |
| ENSSSCG00000020835 | EIF4EBP3 | 2 | 142303859 | 142305716 |  |
| ENSSSCG00000014365 | SRA1 | 2 | 142307012 | 142320570 |  |
| ENSSSCG00000014366 | APBB3 | 2 | 142314076 | 142323475 |  |
| ENSSSCG00000014367 | SLC35A4 | 2 | 142320952 | 142325434 |  |
| ENSSSCG00000014368 |  | 2 | 142326316 | 142339086 |  |
| ENSSSCG00000014369 | CD14 | 2 | 142346211 | 142349349 |  |
| ENSSSCG00000014370 | TMCO6 | 2 | 142351982 | 142361521 |  |
| ENSSSCG00000014371 |  | 2 | 142359006 | 142361131 |  |
| ENSSSCG00000014372 | IK | 2 | 142361187 | 142378745 |  |
| ENSSSCG00000032062 | WDR55 | 2 | 142378462 | 142384677 |  |
| ENSSSCG00000014374 | DND1 | 2 | 142384994 | 142388440 |  |
| ENSSSCG00000014375 | HARS | 2 | 142386733 | 142401136 |  |
| ENSSSCG00000014376 | HARS2 | 2 | 142401284 | 142417004 |  |
| ENSSSCG00000029158 | ZMAT2 | 2 | 142411086 | 142417154 |  |
| ENSSSCG00000023841 | RF00006 | 2 | 142420075 | 142420169 |  |
| ENSSSCG00000026471 | PCDHA1 | 2 | 142460178 | 142465237 |  |
| ENSSSCG00000022218 |  | 2 | 142462020 | 142466115 |  |
| ENSSSCG00000029281 | PCDHAC2 | 2 | 142472588 | 142688089 |  |
| ENSSSCG00000039859 |  | 2 | 142474432 | 142475339 |  |
| ENSSSCG00000039576 |  | 2 | 142481882 | 142485771 |  |
| ENSSSCG00000025603 | PCDHA4 | 2 | 142486346 | 142490452 |  |
| ENSSSCG00000038309 |  | 2 | 142486973 | 142492354 |  |
| ENSSSCG00000029442 |  | 2 | 142505136 | 142527509 |  |
| ENSSSCG00000037515 |  | 2 | 142518978 | 142521265 |  |
| ENSSSCG00000030257 |  | 2 | 142526792 | 142527319 |  |
| ENSSSCG00000040211 |  | 2 | 142532256 | 142534002 |  |
| ENSSSCG00000024998 | PCDHA11 | 2 | 142533601 | 142547107 |  |
| ENSSSCG00000031256 |  | 2 | 142540219 | 142544950 |  |
| ENSSSCG00000028030 | PCDHA13 | 2 | 142547430 | 142553513 |  |
| ENSSSCG00000014377 | PCDHB1 | 2 | 142741988 | 142744443 |  |
| ENSSSCG00000014379 | PCDHB2 | 2 | 142802270 | 142809530 |  |
| ENSSSCG00000036597 |  | 2 | 142806530 | 142818523 |  |
| ENSSSCG00000014378 |  | 2 | 142817677 | 142820079 |  |
| ENSSSCG00000014380 |  | 2 | 142830525 | 142832881 |  |
| ENSSSCG00000032454 |  | 2 | 142831031 | 142831228 |  |
| ENSSSCG00000036516 |  | 2 | 142843311 | 142861692 |  |
| ENSSSCG00000035917 |  | 2 | 142856492 | 142856650 |  |
| ENSSSCG00000024873 | PCDHB6 | 2 | 142870047 | 142872452 |  |
| ENSSSCG00000021414 |  | 2 | 142875088 | 142877496 |  |
| ENSSSCG00000036961 | PCDHB7 | 2 | 142882725 | 142885139 |  |
| ENSSSCG00000036818 |  | 2 | 142889513 | 142893535 |  |
| ENSSSCG00000027852 |  | 2 | 142892832 | 142895246 |  |
| ENSSSCG00000040956 |  | 2 | 142902520 | 142904448 |  |
| ENSSSCG00000037983 |  | 2 | 142903026 | 142903223 |  |
| ENSSSCG00000036348 | PCDHB15 | 2 | 142908041 | 142915076 |  |
| ENSSSCG00000027249 | SLC25A2 | 2 | 142944078 | 142944980 |  |
| ENSSSCG00000028168 | TAF7 | 2 | 142947380 | 142952518 |  |
| ENSSSCG00000027371 | PCDHGC4 | 2 | 142966008 | 143156552 |  |
| ENSSSCG00000037761 |  | 2 | 142971680 | 142973546 |  |
| ENSSSCG00000031384 | PCDHGA2 | 2 | 142977983 | 142980449 |  |
| ENSSSCG00000033765 | PCDHGA3 | 2 | 142983077 | 142985628 |  |
| ENSSSCG00000025141 | PCDHGB1 | 2 | 142989140 | 142991575 |  |
| ENSSSCG00000038245 |  | 2 | 142989797 | 142990120 |  |
| ENSSSCG00000040315 | PCDHGA7 | 2 | 143021188 | 143023691 |  |
| ENSSSCG00000024762 | PCDHGB4 | 2 | 143026234 | 143028644 |  |
| ENSSSCG00000021326 | PCDHGA8 | 2 | 143031697 | 143034197 |  |
| ENSSSCG00000023485 | PCDHGB5 | 2 | 143036876 | 143039295 |  |
| ENSSSCG00000032137 | PCDHGA9 | 2 | 143041317 | 143156550 |  |
| ENSSSCG00000031533 | PCDHGB6 | 2 | 143046030 | 143048478 |  |
| ENSSSCG00000021051 | PCDHGA10 | 2 | 143050428 | 143060768 |  |
| ENSSSCG00000023932 | PCDHGB7 | 2 | 143054906 | 143057334 |  |
| ENSSSCG00000028382 |  | 2 | 143067729 | 143071262 |  |
| ENSSSCG00000014385 | PCDHGC5 | 2 | 143135591 | 143156552 |  |
| ENSSSCG00000035062 |  | 2 | 143169308 | 143171623 |  |
| ENSSSCG00000014387 |  | 2 | 143224084 | 143270994 |  |
| ENSSSCG00000014388 | HDAC3 | 2 | 143273190 | 143287905 |  |
| ENSSSCG00000014390 | FCHSD1 | 2 | 143287971 | 143301844 |  |
| ENSSSCG00000014389 | RELL2 | 2 | 143287973 | 143292833 |  |
| ENSSSCG00000014391 | ARAP3 | 2 | 143304075 | 143325905 |  |
| ENSSSCG00000022289 | PCDH1 | 2 | 143475942 | 143506105 |  |
| ENSSSCG00000014396 | DELE1 | 2 | 143527860 | 143546486 |  |
| ENSSSCG00000014395 | PCDH12 | 2 | 143548215 | 143564429 |  |
| ENSSSCG00000014394 | RNF14 | 2 | 143574950 | 143595933 |  |
| ENSSSCG00000038940 | GNPDA1 | 2 | 143600827 | 143616051 |  |
| ENSSSCG00000025295 | NDFIP1 | 2 | 143629778 | 143734600 |  |
| ENSSSCG00000036322 | SPRY4 | 2 | 143877340 | 143891285 |  |
| ENSSSCG00000037711 | RF02038 | 2 | 143884827 | 143884916 |  |
| ENSSSCG00000039090 | RF02039 | 2 | 143885002 | 143885119 |  |
| ENSSSCG00000020485 | RF00020 | 2 | 143967991 | 143968102 |  |
| ENSSSCG00000024954 | FGF1 | 2 | 144133407 | 144241374 |  |
| ENSSSCG00000019432 | RF00340 | 2 | 144166752 | 144166879 |  |
| ENSSSCG00000014399 | ARHGAP26 | 2 | 144301192 | 144778086 |  |
| ENSSSCG00000014400 |  | 2 | 144489665 | 144490146 |  |
| ENSSSCG00000014401 | NR3C1 | 2 | 144822078 | 144956456 |  |
| ENSSSCG00000031846 |  | 2 | 144907626 | 144911544 |  |
| ENSSSCG00000014406 | PRELID2 | 2 | 147109586 | 147191803 | **LPS-G** |
| ENSSSCG00000036769 | RF00026 | 2 | 147221320 | 147221426 |  |
| ENSSSCG00000040306 | GRXCR2 | 2 | 147222643 | 147232250 |  |
| ENSSSCG00000014410 | SH3RF2 | 2 | 147292177 | 147411514 |  |
| ENSSSCG00000014408 | PLAC8L1 | 2 | 147438994 | 147463019 |  |
| ENSSSCG00000014411 | LARS | 2 | 147480336 | 147547298 |  |
| ENSSSCG00000014414 |  | 2 | 147569394 | 147641060 |  |
| ENSSSCG00000014412 | POU4F3 | 2 | 147693014 | 147694345 |  |
| ENSSSCG00000019633 | RF00026 | 2 | 147769292 | 147769398 |  |
| ENSSSCG00000014416 | TCERG1 | 2 | 147792034 | 147852461 |  |
| ENSSSCG00000034305 | RF02271 | 2 | 147851954 | 147852108 |  |
| ENSSSCG00000014415 | GPR151 | 2 | 147856604 | 147857869 |  |
| ENSSSCG00000031406 |  | 2 | 147919774 | 148241614 |  |
| ENSSSCG00000028976 | PPP2R2B | 2 | 147937442 | 148411731 |  |
| ENSSSCG00000014418 | STK32A | 2 | 148565634 | 148656043 |  |
| ENSSSCG00000036549 | DPYSL3 | 2 | 148662051 | 148855649 |  |
| ENSSSCG00000014420 | JAKMIP2 | 2 | 148859418 | 149045932 |  |
| ENSSSCG00000032774 |  | 2 | 149070089 | 149091953 |  |
| ENSSSCG00000022352 | RF00156 | 2 | 149072070 | 149072194 |  |
| ENSSSCG00000034511 | SPINK1 | 2 | 149072368 | 149080366 |  |
| ENSSSCG00000024111 | SCGB3A2 | 2 | 149120665 | 149123915 |  |
| ENSSSCG00000034399 | C5orf46 | 2 | 149146583 | 149151429 |  |
| ENSSSCG00000014422 | SPINK5 | 2 | 149311084 | 149375733 |  |
| ENSSSCG00000038182 |  | 2 | 149373920 | 149409847 |  |
| ENSSSCG00000033173 | SPINK14 | 2 | 149414655 | 149423768 |  |
| ENSSSCG00000039674 | SPINK6 | 2 | 149470402 | 149486649 |  |
| ENSSSCG00000036834 |  | 2 | 149525155 | 149528679 |  |
| ENSSSCG00000037658 |  | 2 | 149543504 | 149575793 |  |
| ENSSSCG00000029257 |  | 2 | 149556827 | 149669308 |  |
| ENSSSCG00000022035 | SPINK7 | 2 | 149611734 | 149615042 |  |
| ENSSSCG00000014426 | SPINK9 | 2 | 149630230 | 149634973 |  |
| ENSSSCG00000014427 | FBXO38 | 2 | 149674292 | 149722865 |  |
| ENSSSCG00000014428 | HTR4 | 2 | 149731034 | 149912495 |  |
| ENSSSCG00000001991 | DHRS1 | 7 | 74992115 | 75002151 | **KLH-G** |
| ENSSSCG00000001993 | TGM1 | 7 | 75021788 | 75045153 |  |
| ENSSSCG00000001992 | RABGGTA | 7 | 75022122 | 75030070 |  |
| ENSSSCG00000026947 | GMPR2 | 7 | 75056745 | 75070084 |  |
| ENSSSCG00000001994 | TINF2 | 7 | 75056804 | 75067918 |  |
| ENSSSCG00000031938 | NEDD8 | 7 | 75070161 | 75085899 |  |
| ENSSSCG00000033006 |  | 7 | 75087471 | 75093792 |  |
| ENSSSCG00000033432 | CHMP4A | 7 | 75089405 | 75093793 |  |
| ENSSSCG00000001999 |  | 7 | 75089441 | 75118735 |  |
| ENSSSCG00000025470 | TSSK4 | 7 | 75091250 | 75104189 |  |
| ENSSSCG00000002000 | IPO4 | 7 | 75104496 | 75118735 |  |
| ENSSSCG00000002001 | REC8 | 7 | 75118744 | 75139630 |  |
| ENSSSCG00000002002 | IRF9 | 7 | 75133696 | 75139230 |  |
| ENSSSCG00000002003 |  | 7 | 75140205 | 75159495 |  |
| ENSSSCG00000002004 |  | 7 | 75153774 | 75159030 |  |
| ENSSSCG00000002005 | EMC9 | 7 | 75159236 | 75161829 |  |
| ENSSSCG00000002006 | PSME1 | 7 | 75160765 | 75164810 |  |
| ENSSSCG00000002007 | FITM1 | 7 | 75161831 | 75168634 |  |
| ENSSSCG00000002008 | DCAF11 | 7 | 75166965 | 75181142 |  |
| ENSSSCG00000002009 | PCK2 | 7 | 75187197 | 75197638 |  |
| ENSSSCG00000002010 | NRL | 7 | 75199196 | 75210605 |  |
| ENSSSCG00000002011 | CPNE6 | 7 | 75213986 | 75219031 |  |
| ENSSSCG00000002012 | CARMIL3 | 7 | 75223075 | 75241264 |  |
| ENSSSCG00000002013 | DHRS4 | 7 | 75241058 | 75255701 |  |
| ENSSSCG00000002014 | JPH4 | 7 | 75522793 | 75532962 |  |
| ENSSSCG00000002015 | AP1G2 | 7 | 75530033 | 75549488 |  |
| ENSSSCG00000002016 | THTPA | 7 | 75545973 | 75552904 |  |
| ENSSSCG00000040339 | ZFHX2 | 7 | 75572547 | 75586132 |  |
| ENSSSCG00000002020 |  | 7 | 75621342 | 75629447 |  |
| ENSSSCG00000002029 | MYH7 | 7 | 75650847 | 75704483 |  |
| ENSSSCG00000019225 | ssc-mir-208b | 7 | 75667786 | 75667865 |  |
| ENSSSCG00000031822 |  | 7 | 75669445 | 75672321 |  |
| ENSSSCG00000019541 | MIR208A | 7 | 75697180 | 75697262 |  |
| ENSSSCG00000040606 |  | 7 | 75699457 | 75704057 |  |
| ENSSSCG00000002028 | CMTM5 | 7 | 75707133 | 75710017 |  |
| ENSSSCG00000028427 | IL25 | 7 | 75710996 | 75714002 |  |
| ENSSSCG00000002026 | EFS | 7 | 75722183 | 75731592 |  |
| ENSSSCG00000002025 | SLC22A17 | 7 | 75734875 | 75741128 |  |
| ENSSSCG00000002023 | PABPN1 | 7 | 75750330 | 75770723 |  |
| ENSSSCG00000028190 | HOMEZ | 7 | 75752418 | 75798026 |  |
| ENSSSCG00000032977 | PPP1R3E | 7 | 75776720 | 75782706 |  |
| ENSSSCG00000026237 | RNF212B | 7 | 75799635 | 75829619 |  |
| ENSSSCG00000002032 | SLC7A8 | 7 | 75854309 | 75922330 |  |
| ENSSSCG00000023430 | RF00026 | 7 | 75897669 | 75897774 |  |
| ENSSSCG00000002033 | CEBPE | 7 | 75927662 | 75929769 |  |
| ENSSSCG00000002035 | C14orf119 | 7 | 75947771 | 75951806 |  |
| ENSSSCG00000002034 | ACIN1 | 7 | 75951053 | 75990083 |  |
| ENSSSCG00000002037 | CDH24 | 7 | 75990130 | 76001229 |  |
| ENSSSCG00000002038 | PSMB11 | 7 | 76005814 | 76006689 |  |
| ENSSSCG00000002036 |  | 7 | 76014516 | 76058891 |  |
| ENSSSCG00000031452 | C14orf93 | 7 | 76033771 | 76059798 |  |
| ENSSSCG00000029201 | AJUBA | 7 | 76063018 | 76072851 |  |
| ENSSSCG00000033178 |  | 7 | 76085642 | 76099174 |  |
| ENSSSCG00000030045 | PRMT5 | 7 | 76110729 | 76124944 |  |
| ENSSSCG00000026700 | RBM23 | 7 | 76120871 | 76136596 |  |
| ENSSSCG00000031874 | REM2 | 7 | 76141680 | 76148030 |  |
| ENSSSCG00000034465 | LRP10 | 7 | 76149307 | 76158905 |  |
| ENSSSCG00000002039 | MMP14 | 7 | 76174503 | 76186094 |  |
| ENSSSCG00000002041 | SLC7A7 | 7 | 76176590 | 76236149 |  |
| ENSSSCG00000002040 | MRPL52 | 7 | 76187454 | 76191616 |  |
| ENSSSCG00000002042 | OXA1L | 7 | 76230906 | 76266427 |  |
| ENSSSCG00000038303 |  | 7 | 76321059 | 76321985 |  |
| ENSSSCG00000033846 |  | 7 | 76334870 | 76335796 |  |
| ENSSSCG00000002045 |  | 7 | 76362992 | 76363747 |  |
| ENSSSCG00000002052 | OR6J1 | 7 | 76392243 | 76393256 |  |
| ENSSSCG00000002051 | ABHD4 | 7 | 76397084 | 76431923 |  |
| ENSSSCG00000002050 | DAD1 | 7 | 76431964 | 76457890 |  |
| ENSSSCG00000033721 |  | 7 | 76466340 | 76536951 |  |
| ENSSSCG00000033704 |  | 7 | 76500442 | 76500501 |  |
| ENSSSCG00000039980 | TRAV26-2 | 7 | 76515620 | 76851275 |  |
| ENSSSCG00000031641 |  | 7 | 76517997 | 76518056 |  |
| ENSSSCG00000037657 |  | 7 | 76536247 | 76536309 |  |
| ENSSSCG00000031495 | TRDV3 | 7 | 76551645 | 76551935 |  |
| ENSSSCG00000022512 | TRDC | 7 | 76552549 | 76673728 |  |
| ENSSSCG00000002053 |  | 7 | 76649949 | 76650227 |  |
| ENSSSCG00000034250 |  | 7 | 76672770 | 76673051 |  |
| ENSSSCG00000002055 |  | 7 | 76720812 | 76721102 |  |
| ENSSSCG00000002056 | TRAV41 | 7 | 76759754 | 76760023 |  |
| ENSSSCG00000036355 | TRAV39 | 7 | 76783494 | 76783766 |  |
| ENSSSCG00000038317 |  | 7 | 76801170 | 76801451 |  |
| ENSSSCG00000040210 |  | 7 | 76805174 | 76805458 |  |
| ENSSSCG00000037611 |  | 7 | 76810605 | 76810872 |  |
| ENSSSCG00000027788 | TRAV36DV7 | 7 | 76832222 | 76833363 |  |
| ENSSSCG00000037203 |  | 7 | 76839986 | 76878916 |  |
| ENSSSCG00000022057 |  | 7 | 76857462 | 76857749 |  |
| ENSSSCG00000034930 | TRAV29DV5 | 7 | 76865007 | 76866130 |  |
| ENSSSCG00000002062 |  | 7 | 76871857 | 76872126 |  |
| ENSSSCG00000031200 |  | 7 | 76885002 | 76885274 |  |
| ENSSSCG00000040505 |  | 7 | 76893180 | 76893446 |  |
| ENSSSCG00000040308 |  | 7 | 76901682 | 76901957 |  |
| ENSSSCG00000031632 |  | 7 | 76924600 | 76924884 |  |
| ENSSSCG00000033104 |  | 7 | 76929473 | 77221300 |  |
| ENSSSCG00000039621 |  | 7 | 76944995 | 76975395 |  |
| ENSSSCG00000036249 |  | 7 | 76953550 | 76953822 |  |
| ENSSSCG00000032286 |  | 7 | 76968215 | 77010914 |  |
| ENSSSCG00000002081 |  | 7 | 76978175 | 76979901 |  |
| ENSSSCG00000021202 |  | 7 | 76986630 | 76989325 |  |
| ENSSSCG00000036061 |  | 7 | 76995620 | 76995889 |  |
| ENSSSCG00000002090 |  | 7 | 76996506 | 77004698 |  |
| ENSSSCG00000027075 |  | 7 | 77001976 | 77025536 |  |
| ENSSSCG00000033153 |  | 7 | 77019862 | 77020149 |  |
| ENSSSCG00000002077 |  | 7 | 77025066 | 77025251 |  |
| ENSSSCG00000036097 |  | 7 | 77028600 | 77044110 |  |
| ENSSSCG00000040415 |  | 7 | 77051319 | 77056115 |  |
| ENSSSCG00000023624 |  | 7 | 77064758 | 77065033 |  |
| ENSSSCG00000038759 |  | 7 | 77064764 | 77064913 |  |
| ENSSSCG00000002071 |  | 7 | 77066896 | 77067781 |  |
| ENSSSCG00000036734 |  | 7 | 77074274 | 77075505 |  |
| ENSSSCG00000039381 |  | 7 | 77089224 | 77089508 |  |
| ENSSSCG00000040640 |  | 7 | 77112168 | 77112452 |  |
| ENSSSCG00000031215 |  | 7 | 77140539 | 77140808 |  |
| ENSSSCG00000036745 |  | 7 | 77152679 | 77152963 |  |
| ENSSSCG00000032379 |  | 7 | 77157227 | 77157505 |  |
| ENSSSCG00000035163 |  | 7 | 77157236 | 77157502 |  |
| ENSSSCG00000039709 |  | 7 | 77171479 | 77172859 |  |
| ENSSSCG00000035351 |  | 7 | 77182073 | 77182348 |  |
| ENSSSCG00000038909 |  | 7 | 77189980 | 77190270 |  |
| ENSSSCG00000036833 |  | 7 | 77193631 | 77194251 |  |
| ENSSSCG00000032714 |  | 7 | 77204958 | 77205230 |  |
| ENSSSCG00000036550 |  | 7 | 77212867 | 77213151 |  |
| ENSSSCG00000039839 |  | 7 | 77216538 | 77217103 |  |
| ENSSSCG00000033515 |  | 7 | 77227729 | 77228004 |  |
| ENSSSCG00000033825 |  | 7 | 77235542 | 77235826 |  |
| ENSSSCG00000032965 |  | 7 | 77240532 | 77242657 |  |
| ENSSSCG00000034565 |  | 7 | 77249659 | 77249934 |  |
| ENSSSCG00000032246 |  | 7 | 77256610 | 77256882 |  |
| ENSSSCG00000039362 |  | 7 | 77267116 | 77269029 |  |
| ENSSSCG00000031752 |  | 7 | 77275014 | 77277955 |  |
| ENSSSCG00000036513 |  | 7 | 77283912 | 77284631 |  |
| ENSSSCG00000002084 |  | 7 | 77288086 | 77289260 |  |
| ENSSSCG00000036292 |  | 7 | 77290753 | 77291031 |  |
| ENSSSCG00000040548 |  | 7 | 77310953 | 77333092 |  |
| ENSSSCG00000023790 |  | 7 | 77326978 | 77332750 |  |
| ENSSSCG00000032371 | TRAV17 | 7 | 77342001 | 77342575 |  |
| ENSSSCG00000035870 |  | 7 | 77346978 | 77348196 |  |
| ENSSSCG00000036918 |  | 7 | 77355610 | 77357845 |  |
| ENSSSCG00000038885 |  | 7 | 77359149 | 77392648 |  |
| ENSSSCG00000039438 |  | 7 | 77375733 | 77376002 |  |
| ENSSSCG00000002095 | TRAV10 | 7 | 77379415 | 77379693 |  |
| ENSSSCG00000037393 |  | 7 | 77399797 | 77400072 |  |
| ENSSSCG00000002097 |  | 7 | 77406840 | 77407127 |  |
| ENSSSCG00000034169 |  | 7 | 77414784 | 77415059 |  |
| ENSSSCG00000002099 |  | 7 | 77419633 | 77420201 |  |
| ENSSSCG00000002100 | TRAV9-2 | 7 | 77427395 | 77428069 |  |
| ENSSSCG00000034110 |  | 7 | 77432711 | 77436843 |  |
| ENSSSCG00000036635 | TRAV6 | 7 | 77445856 | 77447335 |  |
| ENSSSCG00000034652 | TRAV5 | 7 | 77449939 | 77450211 |  |
| ENSSSCG00000022477 |  | 7 | 77454584 | 77454853 |  |
| ENSSSCG00000002078 |  | 7 | 77457621 | 77457902 |  |
| ENSSSCG00000035110 | TRAV4 | 7 | 77463763 | 77464032 |  |
| ENSSSCG00000032592 | TRAV3 | 7 | 77481930 | 77487706 |  |
| ENSSSCG00000031604 |  | 7 | 77487012 | 77487269 |  |
| ENSSSCG00000002105 | OR4E1 | 7 | 77517445 | 77518398 |  |
| ENSSSCG00000002106 | OR4E2 | 7 | 77528184 | 77529122 |  |
| ENSSSCG00000034872 |  | 7 | 77538256 | 77538525 |  |
| ENSSSCG00000039232 | OR10G2 | 7 | 77547117 | 77548071 |  |
| ENSSSCG00000026253 |  | 7 | 77575271 | 77576199 |  |
| ENSSSCG00000026156 | OR10G3 | 7 | 77592790 | 77593728 |  |
| ENSSSCG00000022705 | SALL2 | 7 | 77638836 | 77653376 |  |
| ENSSSCG00000024445 | METTL3 | 7 | 77661946 | 77690107 |  |
| ENSSSCG00000029203 | TOX4 | 7 | 77677413 | 77698956 |  |
| ENSSSCG00000029544 | RAB2B | 7 | 77689551 | 77716772 |  |
| ENSSSCG00000002127 | CHD8 | 7 | 77719517 | 77780205 |  |
| ENSSSCG00000030175 | RF00377 | 7 | 77770851 | 77770960 |  |
| ENSSSCG00000021463 | RF00377 | 7 | 77775621 | 77775732 |  |
| ENSSSCG00000022900 | SUPT16H | 7 | 77781322 | 77817695 |  |
| ENSSSCG00000002129 | RPGRIP1 | 7 | 77814776 | 77887875 |  |
| ENSSSCG00000033147 |  | 7 | 77895630 | 77895817 |  |
| ENSSSCG00000038947 | HNRNPC | 7 | 77921113 | 77973088 |  |
| ENSSSCG00000032876 | OR5AU1 | 7 | 77996714 | 77997715 |  |
| ENSSSCG00000009584 | SEMA4D | 14 | 957081 | 1078564 | **Ave-G** |
| ENSSSCG00000035249 | GADD45G | 14 | 1165165 | 1167124 |  |
| ENSSSCG00000031962 |  | 14 | 1365729 | 1365956 |  |
| ENSSSCG00000034850 | RF00026 | 14 | 1857573 | 1857674 |  |
| ENSSSCG00000037842 |  | 14 | 1937519 | 1938094 |  |
| ENSSSCG00000031694 | DIRAS2 | 14 | 1968163 | 1968762 |  |
| ENSSSCG00000009589 | SYK | 14 | 2056294 | 2162059 |  |
| ENSSSCG00000009591 | AUH | 14 | 2398460 | 2546372 |  |
| ENSSSCG00000009592 | NFIL3 | 14 | 2588896 | 2603255 |  |
| ENSSSCG00000009593 | ROR2 | 14 | 2804061 | 3035341 |  |
| ENSSSCG00000009594 | SPTLC1 | 14 | 3081972 | 3138103 |  |
| ENSSSCG00000009595 |  | 14 | 3166217 | 3199480 |  |
| ENSSSCG00000033204 |  | 14 | 3295238 | 3501238 |  |
| ENSSSCG00000032926 |  | 17 | 7256805 | 7261630 | **PDG-G and Ave-G** |
| ENSSSCG00000032493 | TRIML1 | 17 | 7284392 | 7290556 |  |
| ENSSSCG00000006997 | TRIML2 | 17 | 7309591 | 7325118 |  |
| ENSSSCG00000035444 | ZFP42 | 17 | 7402158 | 7403157 |  |
| ENSSSCG00000023375 | RF00026 | 17 | 8201832 | 8201934 |  |
| ENSSSCG00000007000 | FAT1 | 17 | 8466099 | 8579924 |  |
| ENSSSCG00000007002 | MTNR1A | 17 | 8596928 | 8617657 |  |
| ENSSSCG00000007003 |  | 17 | 8640378 | 8665327 |  |
| ENSSSCG00000007005 |  | 17 | 8795539 | 8842028 |  |
| ENSSSCG00000038695 |  | 17 | 8959711 | 8962539 |  |
| ENSSSCG00000031677 |  | 17 | 8965937 | 8971000 |  |
| ENSSSCG00000007172 | TMC2 | 17 | 32977995 | 33035318 | **KLH-G** |
| ENSSSCG00000034871 |  | 17 | 33069067 | 33071031 |  |
| ENSSSCG00000040857 |  | 17 | 33086005 | 33094841 |  |
| ENSSSCG00000007178 |  | 17 | 33088454 | 33092354 |  |
| ENSSSCG00000034528 | TGM6 | 17 | 33108569 | 33140139 |  |
| ENSSSCG00000026043 | TGM3 | 17 | 33191909 | 33236609 |  |
| ENSSSCG00000007179 | STK35 | 17 | 33367349 | 33412281 |  |
| ENSSSCG00000037684 | PDYN | 17 | 33504869 | 33521903 |  |
| ENSSSCG00000028461 |  | 17 | 33566270 | 33611368 |  |
| ENSSSCG00000027665 |  | 17 | 33735160 | 33821214 |  |
| ENSSSCG00000035816 |  | 17 | 33747089 | 33774285 |  |
| ENSSSCG00000007186 |  | 17 | 33761282 | 33814043 |  |
| ENSSSCG00000007181 |  | 17 | 33767561 | 33779559 |  |
| ENSSSCG00000007185 | SIRPB2 | 17 | 33850396 | 33865461 |  |
| ENSSSCG00000007187 | NSFL1C | 17 | 33869506 | 33894109 |  |
| ENSSSCG00000007188 |  | 17 | 33910040 | 33935209 |  |
| ENSSSCG00000007189 | SDCBP2 | 17 | 33968086 | 33989694 |  |
| ENSSSCG00000039392 | SNPH | 17 | 33989696 | 33998099 |  |
| ENSSSCG00000007191 | RAD21L1 | 17 | 34035526 | 34064189 |  |
| ENSSSCG00000032426 |  | 17 | 34039224 | 34039511 |  |
| ENSSSCG00000007192 | C20orf202 | 17 | 34083706 | 34085955 |  |
| ENSSSCG00000038854 | PSMF1 | 17 | 34098991 | 34164748 |  |
| ENSSSCG00000035262 | TMEM74B | 17 | 34103297 | 34103994 |  |
| ENSSSCG00000007197 | RSPO4 | 17 | 34268783 | 34309982 |  |
| ENSSSCG00000034004 | ssc-mir-9798 | 17 | 34340792 | 34340874 |  |
| ENSSSCG00000007198 | ANGPT4 | 17 | 34348356 | 34395821 |  |
| ENSSSCG00000007200 | FAM110A | 17 | 34424529 | 34437700 |  |
| ENSSSCG00000007199 | SLC52A3 | 17 | 34459359 | 34481128 |  |
| ENSSSCG00000039028 | SCRT2 | 17 | 34558247 | 34570167 |  |
| ENSSSCG00000023298 | SRXN1 | 17 | 34582410 | 34589300 |  |
| ENSSSCG00000036940 | TCF15 | 17 | 34626656 | 34635194 |  |
| ENSSSCG00000007203 |  | 17 | 34693068 | 34748361 |  |
| ENSSSCG00000036081 | TBC1D20 | 17 | 34760299 | 34779701 |  |
| ENSSSCG00000039862 | TRIB3 | 17 | 34777324 | 34815987 |  |
| ENSSSCG00000007206 | RBCK1 | 17 | 34782052 | 34801230 |  |
| ENSSSCG00000031519 | NRSN2 | 17 | 34836339 | 34845073 |  |
| ENSSSCG00000021920 | SOX12 | 17 | 34857022 | 34857960 |  |
| ENSSSCG00000007211 | ZCCHC3 | 17 | 34884280 | 34885488 |  |
| ENSSSCG00000007212 | C20orf96 | 17 | 34893005 | 34916647 |  |
| ENSSSCG00000038183 |  | 17 | 34922244 | 34924314 |  |
| ENSSSCG00000007218 | DEFB129 | 17 | 34938759 | 34942423 |  |
| ENSSSCG00000034080 | DEFB128 | 17 | 34963686 | 34968203 |  |
| ENSSSCG00000035759 | DEFB127 | 17 | 34973644 | 34975946 |  |
| ENSSSCG00000007217 | DEFB125 | 17 | 35003868 | 35010677 |  |
| ENSSSCG00000007216 |  | 17 | 35028635 | 35031557 |  |
| ENSSSCG00000033857 |  | 17 | 35040170 | 35056835 |  |
| ENSSSCG00000007224 |  | 17 | 35043632 | 35044109 |  |
| ENSSSCG00000007215 | DEFB116 | 17 | 35064541 | 35066964 |  |
| ENSSSCG00000030743 |  | 17 | 35105715 | 35107792 |  |
| ENSSSCG00000007221 |  | 17 | 35131681 | 35142138 |  |
| ENSSSCG00000028629 |  | 17 | 35154178 | 35155784 |  |
| ENSSSCG00000007219 |  | 17 | 35167887 | 35173695 |  |
| ENSSSCG00000007226 | DEFB123 | 17 | 35185472 | 35190548 |  |
| ENSSSCG00000030733 | DEFB124 | 17 | 35200608 | 35204092 |  |
| ENSSSCG00000028529 | REM1 | 17 | 35208360 | 35219959 |  |
| ENSSSCG00000037809 | ssc-mir-7140 | 17 | 35209986 | 35210046 |  |
| ENSSSCG00000007228 | HM13 | 17 | 35248516 | 35291930 |  |
| ENSSSCG00000035357 | BCL2L1 | 17 | 35315475 | 35415778 |  |
| ENSSSCG00000037016 | ID1 | 17 | 35316620 | 35322100 |  |
| ENSSSCG00000038384 | COX4I2 | 17 | 35340572 | 35346087 |  |
| ENSSSCG00000036073 |  | 17 | 35366304 | 35371478 |  |
| ENSSSCG00000007235 | TPX2 | 17 | 35415587 | 35485071 |  |
| ENSSSCG00000007231 | MYLK2 | 17 | 35499823 | 35513092 |  |
| ENSSSCG00000033613 | FOXS1 | 17 | 35523413 | 35537506 |  |
| ENSSSCG00000007232 | DUSP15 | 17 | 35538491 | 35550577 |  |
| ENSSSCG00000007236 | TTLL9 | 17 | 35558301 | 35609033 |  |
| ENSSSCG00000007237 | PDRG1 | 17 | 35607954 | 35616546 |  |
| ENSSSCG00000007238 | XKR7 | 17 | 35632287 | 35658305 |  |
| ENSSSCG00000007239 | CCM2L | 17 | 35671064 | 35693269 |  |
| ENSSSCG00000007240 | HCK | 17 | 35708164 | 35754910 |  |
| ENSSSCG00000007246 | TM9SF4 | 17 | 35765098 | 35822357 |  |
| ENSSSCG00000032676 |  | 17 | 35837370 | 35838771 |  |
| ENSSSCG00000007243 | PLAGL2 | 17 | 35840633 | 35856472 |  |
| ENSSSCG00000007244 | POFUT1 | 17 | 35856573 | 35882964 |  |
| ENSSSCG00000007247 | KIF3B | 17 | 35914214 | 35961687 |  |
| ENSSSCG00000007248 | ASXL1 | 17 | 35985535 | 36068178 |  |
| ENSSSCG00000007469 | PTPN1 | 17 | 52017079 | 52087093 | **KLH-G** |
| ENSSSCG00000007470 | RIPOR3 | 17 | 52088053 | 52162554 |  |
| ENSSSCG00000027206 | PARD6B | 17 | 52156340 | 52213850 |  |
| ENSSSCG00000007472 | BCAS4 | 17 | 52239399 | 52304259 |  |
| ENSSSCG00000007473 | ADNP | 17 | 52317366 | 52355467 |  |
| ENSSSCG00000034952 | DPM1 | 17 | 52350235 | 52377275 |  |
| ENSSSCG00000007475 | MOCS3 | 17 | 52377475 | 52380250 |  |
| ENSSSCG00000007476 | KCNG1 | 17 | 52421352 | 52444842 |  |
| ENSSSCG00000038209 |  | 17 | 52479020 | 52480266 |  |
| ENSSSCG00000007477 | NFATC2 | 17 | 52743570 | 52907428 |  |
| ENSSSCG00000035844 |  | 17 | 52936065 | 52936242 |  |
| ENSSSCG00000007478 | ATP9A | 17 | 52936169 | 53072605 |  |
| ENSSSCG00000007479 | SALL4 | 17 | 53079636 | 53103023 |  |
| ENSSSCG00000020261 | RF00001 | 17 | 53096621 | 53096708 |  |
| ENSSSCG00000019814 | RF00614 | 17 | 53112165 | 53112299 |  |
| ENSSSCG00000019613 | RF00100 | 17 | 53183200 | 53183508 |  |
| ENSSSCG00000007480 |  | 17 | 53290675 | 53291118 |  |
| ENSSSCG00000007481 | ZFP64 | 17 | 53369885 | 53459586 |  |
| ENSSSCG00000032630 | RF02271 | 17 | 53687686 | 53687868 |  |
| ENSSSCG00000040839 |  | 17 | 53705212 | 54557486 |  |
| ENSSSCG00000007482 |  | 17 | 54371631 | 54374699 |  |
| ENSSSCG00000034702 |  | 17 | 54557574 | 54559861 |  |
| ENSSSCG00000007484 | ZNF217 | 17 | 54605939 | 54641881 |  |
| ENSSSCG00000019767 | RF00100 | 17 | 54799214 | 54799477 |  |
| ENSSSCG00000007485 | BCAS1 | 17 | 54917240 | 55040056 |  |
| ENSSSCG00000020469 | RF00619 | 17 | 54963057 | 54963163 |  |

Supplementary Table 2. The list of potential genes around the significant genomic region windows associated with IgM NAb levels.

| **Gene stable ID** | **Gene name** | **Chromosome** | **Gene start (bp)** | **Gene end (bp)** | **Phenotype associated with GWAS hit regions** |
| --- | --- | --- | --- | --- | --- |
| ENSSSCG00000014406 | PRELID2 | 2 | 147109586 | 147191803 | **KLH-M and LPS-M** |
| ENSSSCG00000036769 | RF00026 | 2 | 147221320 | 147221426 |  |
| ENSSSCG00000040306 | GRXCR2 | 2 | 147222643 | 147232250 |  |
| ENSSSCG00000014410 | SH3RF2 | 2 | 147292177 | 147411514 |  |
| ENSSSCG00000014408 | PLAC8L1 | 2 | 147438994 | 147463019 |  |
| ENSSSCG00000014411 | LARS | 2 | 147480336 | 147547298 |  |
| ENSSSCG00000014414 |  | 2 | 147569394 | 147641060 |  |
| ENSSSCG00000014412 | POU4F3 | 2 | 147693014 | 147694345 |  |
| ENSSSCG00000019633 | RF00026 | 2 | 147769292 | 147769398 |  |
| ENSSSCG00000014416 | TCERG1 | 2 | 147792034 | 147852461 |  |
| ENSSSCG00000034305 | RF02271 | 2 | 147851954 | 147852108 |  |
| ENSSSCG00000014415 | GPR151 | 2 | 147856604 | 147857869 |  |
| ENSSSCG00000031406 |  | 2 | 147919774 | 148241614 |  |
| ENSSSCG00000028976 | PPP2R2B | 2 | 147937442 | 148411731 |  |
| ENSSSCG00000014418 | STK32A | 2 | 148565634 | 148656043 |  |
| ENSSSCG00000036549 | DPYSL3 | 2 | 148662051 | 148855649 |  |
| ENSSSCG00000014420 | JAKMIP2 | 2 | 148859418 | 149045932 |  |
| ENSSSCG00000032774 |  | 2 | 149070089 | 149091953 |  |
| ENSSSCG00000022352 | RF00156 | 2 | 149072070 | 149072194 |  |
| ENSSSCG00000034511 | SPINK1 | 2 | 149072368 | 149080366 |  |
| ENSSSCG00000024111 | SCGB3A2 | 2 | 149120665 | 149123915 |  |
| ENSSSCG00000034399 | C5orf46 | 2 | 149146583 | 149151429 |  |
| ENSSSCG00000014422 | SPINK5 | 2 | 149311084 | 149375733 |  |
| ENSSSCG00000038182 |  | 2 | 149373920 | 149409847 |  |
| ENSSSCG00000033173 | SPINK14 | 2 | 149414655 | 149423768 |  |
| ENSSSCG00000039674 | SPINK6 | 2 | 149470402 | 149486649 |  |
| ENSSSCG00000036834 |  | 2 | 149525155 | 149528679 |  |
| ENSSSCG00000037658 |  | 2 | 149543504 | 149575793 |  |
| ENSSSCG00000029257 |  | 2 | 149556827 | 149669308 |  |
| ENSSSCG00000022035 | SPINK7 | 2 | 149611734 | 149615042 |  |
| ENSSSCG00000014426 | SPINK9 | 2 | 149630230 | 149634973 |  |
| ENSSSCG00000014427 | FBXO38 | 2 | 149674292 | 149722865 |  |
| ENSSSCG00000014428 | HTR4 | 2 | 149731034 | 149912495 |  |
| ENSSSCG00000035036 | TDRD15 | 3 | 117182368 | 117189539 | **Ave-M** |
| ENSSSCG00000008595 | APOB | 3 | 117250096 | 117319868 |  |
| ENSSSCG00000025357 | LDAH | 3 | 117463827 | 117575717 |  |
| ENSSSCG00000028931 | GDF7 | 3 | 117588431 | 117592466 |  |
| ENSSSCG00000030115 | HS1BP3 | 3 | 117609027 | 117648384 |  |
| ENSSSCG00000036290 |  | 3 | 117761700 | 117762460 |  |
| ENSSSCG00000038406 |  | 3 | 117773482 | 117777502 |  |
| ENSSSCG00000008599 |  | 3 | 117799365 | 117811702 |  |
| ENSSSCG00000008600 | PUM2 | 3 | 117857960 | 117965567 |  |
| ENSSSCG00000008601 | SDC1 | 3 | 117986688 | 118011886 |  |
| ENSSSCG00000008602 | LAPTM4A | 3 | 118152587 | 118170279 |  |
| ENSSSCG00000008603 | MATN3 | 3 | 118183007 | 118205558 |  |
| ENSSSCG00000008604 | WDR35 | 3 | 118208863 | 118277853 |  |
| ENSSSCG00000037971 |  | 3 | 118273063 | 118279184 |  |
| ENSSSCG00000008606 | OSR1 | 3 | 118597516 | 118765104 |  |
| ENSSSCG00000040444 |  | 3 | 118900484 | 118900747 |  |
| ENSSSCG00000035476 |  | 3 | 119178316 | 119203329 |  |
| ENSSSCG00000008607 |  | 3 | 119413054 | 119453951 |  |
| ENSSSCG00000020040 | RF00026 | 3 | 119435802 | 119435911 |  |
| ENSSSCG00000008608 | RDH14 | 3 | 119453995 | 119458780 |  |
| ENSSSCG00000031905 | KCNS3 | 3 | 119951097 | 119989366 |  |
| ENSSSCG00000023060 |  | 3 | 119993568 | 119994539 |  |
| ENSSSCG00000024065 | TMTC2 | 5 | 98697938 | 98926844 | **LPS-M** |
| ENSSSCG00000020637 | RF00026 | 5 | 98779661 | 98779764 |  |
| ENSSSCG00000000941 | METTL25 | 5 | 99230284 | 99359244 |  |
| ENSSSCG00000040032 |  | 5 | 99297125 | 99298088 |  |
| ENSSSCG00000038987 | CCDC59 | 5 | 99358244 | 99367980 |  |
| ENSSSCG00000033473 |  | 5 | 99451960 | 99452596 |  |
| ENSSSCG00000000940 | PPFIA2 | 5 | 99818795 | 100279030 |  |
| ENSSSCG00000000939 | ACSS3 | 5 | 100270239 | 100429204 |  |
| ENSSSCG00000040348 | LIN7A | 5 | 100537883 | 100686706 |  |
| ENSSSCG00000019821 | RF00003 | 5 | 100571904 | 100572065 |  |
| ENSSSCG00000039181 |  | 5 | 100751930 | 100782290 |  |
| ENSSSCG00000000937 | MYF5 | 5 | 100753501 | 100755494 |  |
| ENSSSCG00000026533 | MYF6 | 5 | 100760487 | 100764767 |  |
| ENSSSCG00000031716 | PTPRQ | 5 | 100787885 | 101023306 |  |
| ENSSSCG00000020583 | RF00100 | 6 | 6231774 | 6232069 | **KLH-M** |
| ENSSSCG00000034572 |  | 6 | 6254509 | 6271019 |  |
| ENSSSCG00000038221 | HSD17B2 | 6 | 6292794 | 6360707 |  |
| ENSSSCG00000035182 | SDR42E1 | 6 | 6382748 | 6400328 |  |
| ENSSSCG00000002688 | PLCG2 | 6 | 6433143 | 6603090 |  |
| ENSSSCG00000002689 | CMIP | 6 | 6654409 | 6882203 |  |
| ENSSSCG00000002690 | GAN | 6 | 6931564 | 6989675 |  |
| ENSSSCG00000036572 | BCO1 | 6 | 7008689 | 7053147 |  |
| ENSSSCG00000039200 |  | 6 | 7063536 | 7166458 |  |
| ENSSSCG00000040203 |  | 6 | 7171179 | 7184734 |  |
| ENSSSCG00000035388 | C16orf46 | 6 | 7189180 | 7199173 |  |
| ENSSSCG00000032216 |  | 6 | 7209526 | 7223085 |  |
| ENSSSCG00000039216 | CENPN | 6 | 7227990 | 7254896 |  |
| ENSSSCG00000032060 |  | 6 | 7255180 | 7278713 |  |
| ENSSSCG00000038128 | CDYL2 | 6 | 7420683 | 7604949 |  |
| ENSSSCG00000031421 | DYNLRB2 | 6 | 7650493 | 7661308 |  |
| ENSSSCG00000033132 |  | 6 | 7661357 | 7846787 |  |
| ENSSSCG00000038433 | RF00026 | 6 | 7806444 | 7806541 |  |
| ENSSSCG00000040607 | MAF | 6 | 8468112 | 8485223 |  |
| ENSSSCG00000040679 | RF00026 | 6 | 8939681 | 8939787 |  |
| ENSSSCG00000003630 | AGO3 | 6 | 91967494 | 92212455 | **KLH-M** |
| ENSSSCG00000018909 | RF00001 | 6 | 92013348 | 92013462 |  |
| ENSSSCG00000039277 |  | 6 | 92133938 | 92134180 |  |
| ENSSSCG00000003632 |  | 6 | 92230992 | 92232005 |  |
| ENSSSCG00000003633 | TEKT2 | 6 | 92233586 | 92237900 |  |
| ENSSSCG00000003634 | ADPRHL2 | 6 | 92238473 | 92244514 |  |
| ENSSSCG00000033641 | COL8A2 | 6 | 92244005 | 92262808 |  |
| ENSSSCG00000037213 | TRAPPC3 | 6 | 92279643 | 92293561 |  |
| ENSSSCG00000003637 | MAP7D1 | 6 | 92298780 | 92322693 |  |
| ENSSSCG00000019537 | RF00004 | 6 | 92342337 | 92342487 |  |
| ENSSSCG00000029954 | THRAP3 | 6 | 92350072 | 92414023 |  |
| ENSSSCG00000036832 | STK40 | 6 | 92410292 | 92484336 |  |
| ENSSSCG00000028892 |  | 6 | 92412690 | 92429696 |  |
| ENSSSCG00000033892 | EVA1B | 6 | 92430205 | 92432137 |  |
| ENSSSCG00000036709 | OSCP1 | 6 | 92489923 | 92537537 |  |
| ENSSSCG00000038916 | LSM10 | 6 | 92489927 | 92493665 |  |
| ENSSSCG00000034647 | MRPS15 | 6 | 92537599 | 92547750 |  |
| ENSSSCG00000025795 | CSF3R | 6 | 92550075 | 92562431 |  |
| ENSSSCG00000035543 | RF00001 | 6 | 92625585 | 92625691 |  |
| ENSSSCG00000003639 |  | 6 | 92695670 | 92697936 |  |
| ENSSSCG00000003640 | GRIK3 | 6 | 92886610 | 93128582 |  |
| ENSSSCG00000035323 | RF00493 | 6 | 93153306 | 93153368 |  |
| ENSSSCG00000037815 | ZC3H12A | 6 | 93541334 | 93551193 |  |
| ENSSSCG00000034611 | MEAF6 | 6 | 93558899 | 93589066 |  |
| ENSSSCG00000029991 | SNIP1 | 6 | 93597059 | 93612150 |  |
| ENSSSCG00000039034 | DNALI1 | 6 | 93612197 | 93622081 |  |
| ENSSSCG00000031631 | GNL2 | 6 | 93616575 | 93646019 |  |
| ENSSSCG00000032240 | RSPO1 | 6 | 93665749 | 93690660 |  |
| ENSSSCG00000039905 | C1orf109 | 6 | 93741012 | 93750598 |  |
| ENSSSCG00000032909 | CDCA8 | 6 | 93750662 | 93765483 |  |
| ENSSSCG00000038675 | EPHA10 | 6 | 93767723 | 93803855 |  |
| ENSSSCG00000032960 | MANEAL | 6 | 93821734 | 93829456 |  |
| ENSSSCG00000040425 | YRDC | 6 | 93829321 | 93833643 |  |
| ENSSSCG00000038888 | C1orf122 | 6 | 93833760 | 93834916 |  |
| ENSSSCG00000037516 | MTF1 | 6 | 93839200 | 93884417 |  |
| ENSSSCG00000003642 | INPP5B | 6 | 93885459 | 93944384 |  |
| ENSSSCG00000032847 |  | 6 | 93892774 | 93893363 |  |
| ENSSSCG00000019890 | RF00100 | 6 | 93931749 | 93932077 |  |
| ENSSSCG00000003643 | SF3A3 | 6 | 93948335 | 93976158 |  |
| ENSSSCG00000003644 | FHL3 | 6 | 93980635 | 93988573 |  |
| ENSSSCG00000003645 | UTP11 | 6 | 93993703 | 94007039 |  |
| ENSSSCG00000003646 | POU3F1 | 6 | 94026675 | 94028030 |  |
| ENSSSCG00000003648 | RRAGC | 6 | 94785910 | 94814537 |  |
| ENSSSCG00000003650 | GJA9 | 6 | 94815600 | 94817147 |  |
| ENSSSCG00000003651 | RHBDL2 | 6 | 94821350 | 94864101 |  |
| ENSSSCG00000037175 |  | 6 | 94880893 | 94885051 |  |
| ENSSSCG00000003652 | AKIRIN1 | 6 | 94891409 | 94902700 |  |
| ENSSSCG00000003653 | NDUFS5 | 6 | 94912685 | 94919457 |  |
| ENSSSCG00000035413 | RF00026 | 6 | 94930415 | 94930521 |  |
| ENSSSCG00000036526 |  | 6 | 94964178 | 94976744 |  |
| ENSSSCG00000038731 | STX18 | 8 | 5890743 | 6007089 | **LPS-M and Ave-M** |
| ENSSSCG00000024892 | NSG1 | 8 | 5999713 | 6024769 |  |
| ENSSSCG00000008728 | ZBTB49 | 8 | 6048891 | 6081348 |  |
| ENSSSCG00000008729 | LYAR | 8 | 6081332 | 6100104 |  |
| ENSSSCG00000034122 | TMEM128 | 8 | 6114938 | 6127317 |  |
| ENSSSCG00000008731 | OTOP1 | 8 | 6127965 | 6163061 |  |
| ENSSSCG00000008730 | DRD5 | 8 | 6180221 | 6181636 |  |
| ENSSSCG00000031700 | SLC2A9 | 8 | 6192517 | 6367990 |  |
| ENSSSCG00000035462 |  | 8 | 6339413 | 6339864 |  |
| ENSSSCG00000036501 | WDR1 | 8 | 6409441 | 6452516 |  |
| ENSSSCG00000029711 | ZNF518B | 8 | 6559075 | 6576162 |  |
| ENSSSCG00000022448 | CLNK | 8 | 6609051 | 6770094 |  |
| ENSSSCG00000019781 | RF00614 | 8 | 6949502 | 6949631 |  |
| ENSSSCG00000021515 | HS3ST1 | 8 | 7389780 | 7423229 |  |
| ENSSSCG00000019000 | RF00026 | 8 | 7631365 | 7631472 |  |
| ENSSSCG00000039778 |  | 8 | 8251531 | 8254080 |  |
| ENSSSCG00000035927 |  | 8 | 8565900 | 8586045 |  |
| ENSSSCG00000022358 | RAB28 | 8 | 8942648 | 9125674 |  |
| ENSSSCG00000018023 | COX10 | 12 | 58011817 | 58168405 | **Ave-M** |
| ENSSSCG00000039318 | HS3ST3B1 | 12 | 58126163 | 58168404 |  |
| ENSSSCG00000034672 | ssc-mir-9828-1 | 12 | 58566073 | 58566170 |  |
| ENSSSCG00000036258 | ssc-mir-9828-1 | 12 | 58569160 | 58569257 |  |
| ENSSSCG00000033170 | ssc-mir-9828-1 | 12 | 58572254 | 58572351 |  |
| ENSSSCG00000037832 | PMP22 | 12 | 58677409 | 58707972 |  |
| ENSSSCG00000018028 | TEKT3 | 12 | 58734403 | 58789904 |  |
| ENSSSCG00000039792 | CDRT4 | 12 | 58819047 | 58821231 |  |
| ENSSSCG00000018027 |  | 12 | 58873179 | 58923072 |  |
| ENSSSCG00000018026 |  | 12 | 58896154 | 58923072 |  |
| ENSSSCG00000034049 |  | 12 | 58926087 | 58939851 |  |
| ENSSSCG00000018029 |  | 12 | 58964500 | 58981769 |  |
| ENSSSCG00000025254 | ZNF624 | 12 | 58986547 | 59007697 |  |
| ENSSSCG00000018031 | ZNF287 | 12 | 59038737 | 59052727 |  |
| ENSSSCG00000036776 | LRRC75A | 12 | 59094834 | 59132605 |  |
| ENSSSCG00000018462 | RF00571 | 12 | 59132938 | 59133010 |  |
| ENSSSCG00000018456 | RF00277 | 12 | 59133725 | 59133795 |  |
| ENSSSCG00000038146 | RF00277 | 12 | 59134194 | 59134260 |  |
| ENSSSCG00000018032 | TRPV2 | 12 | 59135329 | 59152638 |  |
| ENSSSCG00000018033 | UBB | 12 | 59171405 | 59172094 |  |
| ENSSSCG00000018034 |  | 12 | 59199979 | 59209664 |  |
| ENSSSCG00000037031 |  | 12 | 59201509 | 59276624 |  |
| ENSSSCG00000035103 | PIGL | 12 | 59215184 | 59257344 |  |
| ENSSSCG00000018039 | NCOR1 | 12 | 59273169 | 59391810 |  |
| ENSSSCG00000023747 | TTC19 | 12 | 59392182 | 59418796 |  |
| ENSSSCG00000036482 | ZSWIM7 | 12 | 59418922 | 59435374 |  |
| ENSSSCG00000035051 | ADORA2B | 12 | 59424457 | 59449692 |  |
| ENSSSCG00000034364 | SPECC1 | 12 | 59466786 | 59706556 |  |
| ENSSSCG00000024030 | AKAP10 | 12 | 59731776 | 59789131 |  |
| ENSSSCG00000018045 | ULK2 | 12 | 59806459 | 59878392 |  |
| ENSSSCG00000018044 | ALDH3A1 | 12 | 59900129 | 59905967 |  |
| ENSSSCG00000018042 | SLC47A2 | 12 | 59920770 | 59935894 |  |
| ENSSSCG00000018041 | ALDH3A2 | 12 | 59935973 | 59954159 |  |
| ENSSSCG00000018046 |  | 12 | 59977416 | 60019633 |  |
| ENSSSCG00000033329 | SLC47A1 | 12 | 60019858 | 60056951 |  |
| ENSSSCG00000034609 | RNF112 | 12 | 60114133 | 60123095 |  |
| ENSSSCG00000031076 | MAPK7 | 12 | 60128686 | 60149270 |  |
| ENSSSCG00000036007 | MFAP4 | 12 | 60141072 | 60144627 |  |
| ENSSSCG00000039961 |  | 12 | 60159098 | 60162314 |  |
| ENSSSCG00000039681 | B9D1 | 12 | 60162638 | 60174202 |  |
| ENSSSCG00000040457 | EPN2 | 12 | 60171920 | 60234272 |  |
| ENSSSCG00000018050 |  | 12 | 60261555 | 60281885 |  |
| ENSSSCG00000018049 | SLC5A10 | 12 | 60281801 | 60387837 |  |
| ENSSSCG00000018047 | FAM83G | 12 | 60296750 | 60326434 |  |
| ENSSSCG00000036208 | SHMT1 | 12 | 60414416 | 60436438 |  |
| ENSSSCG00000038189 | SMCR8 | 12 | 60432250 | 60443607 |  |
| ENSSSCG00000035839 | TOP3A | 12 | 60443679 | 60468964 |  |
| ENSSSCG00000037151 | MIEF2 | 12 | 60469035 | 60475952 |  |
| ENSSSCG00000039324 | FLII | 12 | 60475700 | 60488644 |  |
| ENSSSCG00000040859 | LLGL1 | 12 | 60487343 | 60500448 |  |
| ENSSSCG00000035120 | ALKBH5 | 12 | 60504359 | 60529534 |  |
| ENSSSCG00000032392 | MYO15A | 12 | 60533917 | 60581411 |  |
| ENSSSCG00000036328 | DRG2 | 12 | 60582024 | 60598299 |  |
| ENSSSCG00000037336 | GID4 | 12 | 60601003 | 60617459 |  |
| ENSSSCG00000028712 | ATPAF2 | 12 | 60617642 | 60633271 |  |
| ENSSSCG00000037557 | DRC3 | 12 | 60633675 | 60651849 |  |
| ENSSSCG00000038639 | TOM1L2 | 12 | 60652148 | 60729256 |  |
| ENSSSCG00000033626 | SREBF1 | 12 | 60733977 | 60752502 |  |
| ENSSSCG00000033948 | MIR33B | 12 | 60749059 | 60749127 |  |
| ENSSSCG00000038164 | RAI1 | 12 | 60750091 | 60854790 |  |
| ENSSSCG00000018055 | PEMT | 12 | 60892688 | 60919279 |  |
| ENSSSCG00000034044 | RASD1 | 12 | 60922422 | 60926283 |  |
| ENSSSCG00000018053 | MED9 | 12 | 60925162 | 60934004 |  |
| ENSSSCG00000018052 | NT5M | 12 | 60979919 | 60994338 |  |
| ENSSSCG00000018051 | COPS3 | 12 | 60993460 | 61023903 |  |
| ENSSSCG00000009994 | MTMR3 | 14 | 46874972 | 47023489 | **LTA-M** |
| ENSSSCG00000009995 | HORMAD2 | 14 | 47081212 | 47160971 |  |
| ENSSSCG00000040961 | LIF | 14 | 47221486 | 47228273 |  |
| ENSSSCG00000009997 | OSM | 14 | 47242774 | 47246838 |  |
| ENSSSCG00000009998 | CASTOR1 | 14 | 47261911 | 47266734 |  |
| ENSSSCG00000009999 | TBC1D10A | 14 | 47269091 | 47301554 |  |
| ENSSSCG00000010002 | SF3A1 | 14 | 47304808 | 47335398 |  |
| ENSSSCG00000010003 | CCDC157 | 14 | 47341323 | 47349662 |  |
| ENSSSCG00000010005 | RNF215 | 14 | 47351295 | 47357149 |  |
| ENSSSCG00000010006 |  | 14 | 47369658 | 47392873 |  |
| ENSSSCG00000036615 | RF01848 | 14 | 47388883 | 47389095 |  |
| ENSSSCG00000032812 | RF00012 | 14 | 47388884 | 47389095 |  |
| ENSSSCG00000010007 | MTFP1 | 14 | 47392776 | 47395607 |  |
| ENSSSCG00000010008 | SEC14L3 | 14 | 47405809 | 47421277 |  |
| ENSSSCG00000038709 | SEC14L4 | 14 | 47432234 | 47442975 |  |
| ENSSSCG00000010009 | GAL3ST1 | 14 | 47453679 | 47476472 |  |
| ENSSSCG00000010010 | PES1 | 14 | 47478380 | 47495983 |  |
| ENSSSCG00000010011 | TCN2 | 14 | 47496303 | 47517204 |  |
| ENSSSCG00000010012 | SLC35E4 | 14 | 47520089 | 47526704 |  |
| ENSSSCG00000033833 |  | 14 | 47539106 | 47550502 |  |
| ENSSSCG00000010014 | C5orf52 | 14 | 47540359 | 47554963 |  |
| ENSSSCG00000010015 | OSBP2 | 14 | 47557582 | 47736584 |  |
| ENSSSCG00000010016 | MORC2 | 14 | 47754236 | 47799700 |  |
| ENSSSCG00000036935 | RF01882 | 14 | 47801563 | 47801706 |  |
| ENSSSCG00000035042 | RF01883 | 14 | 47801852 | 47801938 |  |
| ENSSSCG00000040413 | RF01891 | 14 | 47803898 | 47804141 |  |
| ENSSSCG00000038425 | RF01892 | 14 | 47804463 | 47804636 |  |
| ENSSSCG00000010017 | SMTN | 14 | 47879317 | 47902699 |  |
| ENSSSCG00000040755 |  | 14 | 47881118 | 47881348 |  |
| ENSSSCG00000029781 | SELENOM | 14 | 47902864 | 47905440 |  |
| ENSSSCG00000030345 | INPP5J | 14 | 47913380 | 47922631 |  |
| ENSSSCG00000010023 | PLA2G3 | 14 | 47923996 | 47928883 |  |
| ENSSSCG00000010024 | RNF185 | 14 | 47946375 | 47979698 |  |
| ENSSSCG00000010025 | LIMK2 | 14 | 47946421 | 48040821 |  |
| ENSSSCG00000010026 | PIK3IP1 | 14 | 48038183 | 48051269 |  |
| ENSSSCG00000010027 | PATZ1 | 14 | 48075348 | 48094136 |  |
| ENSSSCG00000010029 | DRG1 | 14 | 48126802 | 48153152 |  |
| ENSSSCG00000010030 | EIF4ENIF1 | 14 | 48156841 | 48204338 |  |
| ENSSSCG00000035290 |  | 14 | 48210542 | 48266038 |  |
| ENSSSCG00000010031 |  | 14 | 48210570 | 48303508 |  |
| ENSSSCG00000010032 | PISD | 14 | 48303455 | 48381597 |  |
| ENSSSCG00000010033 | PRR14L | 14 | 48351979 | 48418167 |  |
| ENSSSCG00000010034 |  | 14 | 48421875 | 48523593 |  |
| ENSSSCG00000039075 | RF00026 | 14 | 48447408 | 48447514 |  |
| ENSSSCG00000010035 | YWHAH | 14 | 48543691 | 48557966 |  |
| ENSSSCG00000010036 | SLC5A1 | 14 | 48566343 | 48707223 |  |
| ENSSSCG00000010039 | SLC5A4 | 14 | 48708047 | 48741440 |  |
| ENSSSCG00000035443 |  | 14 | 48752438 | 48765031 |  |
| ENSSSCG00000040522 |  | 14 | 48773078 | 48783933 |  |
| ENSSSCG00000030775 |  | 14 | 48800439 | 48800732 |  |
| ENSSSCG00000032042 |  | 14 | 48819533 | 48819829 |  |
| ENSSSCG00000039180 |  | 14 | 48824977 | 48825547 |  |
| ENSSSCG00000032582 |  | 14 | 48828406 | 48830777 |  |
| ENSSSCG00000031037 |  | 14 | 48841940 | 48842417 |  |
| ENSSSCG00000034806 |  | 14 | 48852111 | 48852389 |  |
| ENSSSCG00000038719 |  | 14 | 48858102 | 49012754 |  |
| ENSSSCG00000025508 |  | 14 | 48858148 | 48858548 |  |
| ENSSSCG00000033879 | ZNF280B | 14 | 48861289 | 48877223 |  |
| ENSSSCG00000034651 |  | 14 | 48882955 | 48884625 |  |
| ENSSSCG00000036408 | PRAME | 14 | 48901516 | 48908939 |  |
| ENSSSCG00000039192 |  | 14 | 48919567 | 48920051 |  |
| ENSSSCG00000030830 |  | 14 | 48930112 | 48930399 |  |
| ENSSSCG00000037250 |  | 14 | 48936110 | 48942115 |  |
| ENSSSCG00000037214 |  | 14 | 48943274 | 49010871 |  |
| ENSSSCG00000036324 |  | 14 | 48975238 | 48981238 |  |
| ENSSSCG00000030983 |  | 14 | 48988109 | 48988378 |  |
| ENSSSCG00000010044 |  | 14 | 49001068 | 49001385 |  |
| ENSSSCG00000024860 |  | 14 | 49050166 | 49121385 |  |
| ENSSSCG00000010047 | RSPH14 | 14 | 49123935 | 49184735 |  |
| ENSSSCG00000010046 | GNAZ | 14 | 49132158 | 49174798 |  |
| ENSSSCG00000010048 | RAB36 | 14 | 49189262 | 49200410 |  |
| ENSSSCG00000010052 | BCR | 14 | 49208762 | 49322107 |  |
| ENSSSCG00000010053 |  | 14 | 49332941 | 49464362 |  |
| ENSSSCG00000010054 | ADORA2A | 14 | 49468862 | 49487628 |  |
| ENSSSCG00000010060 | UPB1 | 14 | 49533203 | 49561497 |  |
| ENSSSCG00000010059 | GUCD1 | 14 | 49572096 | 49585748 |  |
| ENSSSCG00000010058 | SNRPD3 | 14 | 49585120 | 49598012 |  |
| ENSSSCG00000010057 | LRRC75B | 14 | 49609347 | 49612867 |  |
| ENSSSCG00000010062 | CABIN1 | 14 | 49615278 | 49787687 |  |
| ENSSSCG00000010056 |  | 14 | 49622122 | 49638061 |  |
| ENSSSCG00000010055 | GGT5 | 14 | 49640937 | 49666293 |  |
| ENSSSCG00000025393 | SUSD2 | 14 | 49673711 | 49683738 |  |
| ENSSSCG00000028151 | RF00001 | 14 | 49696058 | 49696190 |  |
| ENSSSCG00000040195 |  | 14 | 49771588 | 49809626 |  |
| ENSSSCG00000035836 | DDT | 14 | 49790794 | 49807633 |  |
| ENSSSCG00000022566 |  | 14 | 49795092 | 49807648 |  |
| ENSSSCG00000010064 |  | 14 | 49801161 | 49807602 |  |
| ENSSSCG00000010065 | GSTT4 | 14 | 49812129 | 49819025 |  |
| ENSSSCG00000031223 |  | 14 | 49819812 | 49822296 |  |
| ENSSSCG00000010067 | MIF | 14 | 49840302 | 49862322 |  |
| ENSSSCG00000010068 |  | 14 | 49845741 | 49857727 |  |
| ENSSSCG00000034068 |  | 14 | 49847140 | 49863113 |  |
| ENSSSCG00000010070 | SMARCB1 | 14 | 49871056 | 49906028 |  |
| ENSSSCG00000039626 | DERL3 | 14 | 49871218 | 49877672 |  |
| ENSSSCG00000010071 | MMP11 | 14 | 49907694 | 49917471 |  |
| ENSSSCG00000010073 | CHCHD10 | 14 | 49921104 | 49923351 |  |
| ENSSSCG00000010074 |  | 14 | 49924349 | 49926033 |  |
| ENSSSCG00000010076 | ZNF70 | 14 | 49928357 | 49935617 |  |
| ENSSSCG00000010077 |  | 14 | 49951437 | 49960544 |  |
| ENSSSCG00000022467 | VPREB1 | 14 | 49972048 | 49972768 |  |
| ENSSSCG00000026842 | CDH18 | 16 | 8094693 | 8578236 | **KLH-M** |
| ENSSSCG00000032260 |  | 16 | 9952392 | 9957375 |  |
| ENSSSCG00000016799 | CDH12 | 16 | 9985126 | 10267600 |  |
| ENSSSCG00000016800 |  | 16 | 10472810 | 10478021 |  |
